# A Statistically Representative Atlas for Mapping Neuronal Circuits in the *Drosophila* Adult Brain

**DOI:** 10.3389/fninf.2018.00013

**Published:** 2018-03-23

**Authors:** Ignacio Arganda-Carreras, Tudor Manoliu, Nicolas Mazuras, Florian Schulze, Juan E. Iglesias, Katja Bühler, Arnim Jenett, François Rouyer, Philippe Andrey

**Affiliations:** ^1^Institut Jean-Pierre Bourgin, INRA, AgroParisTech, CNRS, Université Paris-Saclay, Versailles, France; ^2^Ikerbasque, Basque Foundation for Science, Bilbao, Spain; ^3^Donostia International Physics Center, Donostia-San Sebastian, Spain; ^4^Institut des Neurosciences Paris-Saclay, Université Paris Sud, CNRS, Université Paris-Saclay, Gif-sur-Yvette, France; ^5^VRVis Zentrum für Virtual Reality und Visualisierung Forschungs-GmbH, Vienna, Austria; ^6^Basque Center on Cognition, Brain and Language, Donostia-San Sebastian, Spain; ^7^Tefor Core Facility, Institut des Neurosciences Paris-Saclay, Université Paris Sud, CNRS, Université Paris-Saclay, Gif-sur-Yvette, France

**Keywords:** *Drosophila* adult brain, anatomical atlas, confocal microscopy, brain mapping, average brain template, diffeomorphic image registration, atlas-based image segmentation

## Abstract

Imaging the expression patterns of reporter constructs is a powerful tool to dissect the neuronal circuits of perception and behavior in the adult brain of *Drosophila*, one of the major models for studying brain functions. To date, several *Drosophila* brain templates and digital atlases have been built to automatically analyze and compare collections of expression pattern images. However, there has been no systematic comparison of performances between alternative atlasing strategies and registration algorithms. Here, we objectively evaluated the performance of different strategies for building adult *Drosophila* brain templates and atlases. In addition, we used state-of-the-art registration algorithms to generate a new group-wise inter-sex atlas. Our results highlight the benefit of statistical atlases over individual ones and show that the newly proposed inter-sex atlas outperformed existing solutions for automated registration and annotation of expression patterns. Over 3,000 images from the Janelia Farm FlyLight collection were registered using the proposed strategy. These registered expression patterns can be searched and compared with a new version of the BrainBaseWeb system and BrainGazer software. We illustrate the validity of our methodology and brain atlas with registration-based predictions of expression patterns in a subset of clock neurons. The described registration framework should benefit to brain studies in *Drosophila* and other insect species.

## 1. Introduction

The fruit fly (*Drosophila melanogaster*) is a well-established model species for studying the neuronal circuits involved in sensory perception (Albert and Göpfert, 2015; Behnia and Desplan, 2015; Joseph and Carlson, 2015) and a wide range of behaviors (Owald and Waddell, 2015; Anderson, 2016; Auer and Benton, 2016; Dubowy and Sehgal, 2017). *Drosophila* has also become an attractive model for brain pathologies and disorders (McGurk et al., 2015; Narayanan and Rothenfluh, 2016), aging and age related decline (Jones and Grotewiel, 2011) or addictions (Kaun et al., 2012). A key advantage of *Drosophila* is the availability of a large collection of transgenic constructs for monitoring or altering neuronal activity (Venken et al., 2011; Sivanantharajah and Zhang, 2015). Thousands of lines have been generated to drive expression of transgenes in specific neuronal populations of the adult brain (Pfeiffer et al., 2008; Jenett et al., 2012; Kvon et al., 2014). Further refinements of the driver-techniques by intersectional strategies now allow to target very small subsets of neurons down to the single-cell level (Luan et al., 2006; Pfeiffer et al., 2010; Gohl et al., 2011; Ting et al., 2011; Dolan et al., 2017). The constructed lines can be characterized by imaging the transgenic expression of fluorescent proteins or using immunohistochemistry against transgenic epitopes (Pfeiffer et al., 2008; Jenett et al., 2012; Nern et al., 2015; Viswanathan et al., 2015). Powerful image processing algorithms and tools are required for the spatially accurate co-analysis of expression patterns acquired on different specimens.

Digital brain atlases are often used to systematically analyze large collections of expression patterns acquired on different specimens (Rein et al., 2002; Jenett et al., 2006; Maye et al., 2006; Knowles and Biggin, 2013). A digital atlas consists of a grayscale template image and an associated anatomical label image. The template is a representative intensity image of a reference pan-neuronal staining (Wagh et al., 2006), where contrast highlights anatomical boundaries between brain regions. The label image assigns any spatial location of the template to a defined anatomical region (Ito et al., 2014). Digital atlases can be used to compare expression patterns between different lines. This registration task is achieved by warping the individual images into the common space of the template, hence standardizing sample position and leveling out inter-individual morphological variations. Atlases can also be used to automatically perform the anatomical annotation of pattern images, for example to quantify transgenic expression in a given region. This segmentation task is achieved by registering the template image onto the pattern images and by propagating the corresponding geometric transformations to the anatomical labels. One benefit of atlas-based image processing is the possibility to run powerful and complex queries on large collections of data through web-based or standalone applications (Bruckner et al., 2009; Chiang et al., 2011; Jenett et al., 2012; Milyaev et al., 2012).

However, various parameters potentially affect the performance of an atlas in the segmentation and registration tasks. Among these, the mathematical model used to represent the geometric transformations between images is critical because it determines the spectrum of morphological variations that can be algorithmically corrected. Early *Drosophila* brain atlases relied on low degree-of-freedom transformations that compensate for variations in position, orientation and scale (Rein et al., 2002). More recently, non-linear deformation models such as B-splines (Rueckert et al., 1999) and thin-plate splines (Bookstein, 1989) were adopted, allowing to capture more complex patterns of morphological variations between specimens (Jefferis et al., 2007; Peng et al., 2011). However, in brain imaging of humans and other mammals, a wider range of transformation models have been proposed for image registration (Gholipour et al., 2007; Sotiras et al., 2013). Comparison between registration methods has shown the superiority of symmetric diffeomorphic registration (Avants et al., 2008) over a number of alternatives (Klein et al., 2009). The potential and benefits of such advanced registration methods has not been investigated yet in the context of *Drosophila* adult brain atlasing, where the targeted resolution should allow the comparison of neuronal processes from individual cells.

In addition, the intensity template and label image of the atlas are also critical parameters impacting on registration and segmentation performance. Two strategies have been adopted in adult *Drosophila* neuroanatomy for building atlases. The first one consists in selecting the intensity and label images from a single individual (Rein et al., 2002; Chiang et al., 2011; Jenett et al., 2012). This introduces a bias toward the morphology of the selected individual. Attempts to minimize this bias have consisted in selecting the most representative individual from a population, for example based on a size criterion or proximity to the average (Rein et al., 2002; Jenett et al., 2006; Chiang et al., 2011). The second atlas-selection strategy consists in building a statistical atlas by averaging images from several individuals after they have been co-registered (Jefferis et al., 2007; Cachero et al., 2010; Yu et al., 2010; Peng et al., 2011; Manton et al., 2014; Costa et al., 2016). The precision of the method used for registering images and its capacity to compensate for anatomical variations between individuals is essential to preserve local contrast when averaging images, since anatomical details condition the precision of the spatial requests that can be performed using an atlas.

Although many strategies have been proposed and evaluated in the last decades for the construction of brain templates in human and other mammalian species (Talairach and Tournoux, 1988; Evans et al., 1993; Mazziotta et al., 1995; Chen et al., 2006; Dogdas et al., 2007; Shattuck et al., 2008), much less has been done in insects. Based on a deformation criterion, strategies for building average atlases of the desert locus brain were compared in Kurylas et al. (2008), but no evaluation for automated segmentation or registration of individual brains was performed. In honeybees, a quantitative comparison study reported superior segmentation performance when using group-wise atlases rather than individual ones (Rohlfing et al., 2004). Apart from these few examples, most current templates of insect brains that have become *de facto* standards have not been quantitatively evaluated for their performances. This can be partially explained by the lack of anatomical images with expert annotations, which could serve as references when evaluating atlas-based segmentations and registrations. For instance, while hundreds of manually labeled brains of mice or humans are publicly accessible (for review, see Dickie et al., 2017), there is only one completely labeled adult *Drosophila* brain available (Ito et al., 2014). Moreover, while the MRI/CT community has proved the superiority of population-based approaches over those based on individual images in the creation of templates in mammal brains (Joshi et al., 2004; Kovacevic et al., 2004; Fonov et al., 2011), these approaches have not been fully translated yet into the insect brain communities.

In the present work, we performed a comprehensive set of experiments to objectively examine the influence of reference brains on atlas performances for both the automated segmentation and registration tasks on *Drosophila* adult brains. Taking benefit from a pre-existing collection of labeled adult *Drosophila* brain images, we compared individual templates to group-wise templates. Furthermore, we generated a new atlas brain built with the most recent image registration technology. Our results highlight the benefits of statistically representative templates in terms of precision and accuracy and show that the newly proposed atlas outperformed the currently available templates. The new *Drosophila* adult brain template was applied and validated using thousands of images from the JFRC FlyLight database (http://flweb.janelia.org/cgi-bin/flew.cgi) as well as newly generated images. The resulting atlas and database of registered images are available for browsing, query, and visualization through a user-friendly web interface (http://fruitfly.tefor.net) and a desktop application providing advanced 3D visualization and querying functionalities.

## 2. Materials and methods

### 2.1. “Würzburg” dataset

Most of the experiments reported here were performed on 44 brain images from the Würzburg dataset (Rein et al., 2002; Jenett et al., 2006), consisting of 22 female and 22 male specimens. These were all adult *Drosophila* brains dissected from 5-day-old flies, stained with nc82 (Wagh et al., 2006) and imaged using a Leica TCS confocal microscope equipped with a Leica 20 × lens with a numerical aperture of 0.7. The original data consisted of 8-bit images of 1, 024 × 1, 024 × 200 voxels with a voxel size of 0.6 × 0.6 × 1.1 μm. A label image was associated with each nc82 image, indicating for each voxel its localization within one out of 14 anatomical regions that had been manually delineated by an expert: left/right medulla, left/right lobula, left/right lobula plate, left/right mushroom body, ellipsoid body, noduli, fan-shaped body, protocerebral bridge, and left/right antennal lobe (see Supplementary Figure 1). These regions are historically the most studied in the insect brain and their overall morphology has been previously described in detail (Rein et al., 2002; Jenett et al., 2006; Ito et al., 2014). They can be unambiguously delineated from the surrounding tissues with the nc82 staining. To obtain nearly cubic voxels and to reduce image processing times, intensity and label images were downsampled by half along the X and Y directions, resulting in a voxel size of 1.2 × 1.2 × 1.1 μm. The corresponding loss of spatial accuracy was negligible compared to the size of *Drosophila* brain structures and to the amplitude of known dimorphic differences (Rein et al., 2002; Jefferis et al., 2007; Cachero et al., 2010).

### 2.2. “Gif” dataset

#### 2.2.1. Experimental animals

Flies were raised on standard yeast/cornmeal/agar medium at 25°C. Young (4–6 days) males were used in all experiments. Three transgenic *gal4*-driver strains (Brand and Perrimon, 1993) were used for double labeling and visualization of specific neurons: *Clk6.1-gal4* (Gummadova et al., 2009), *Pdf-gal4* (Park et al., 2000), and *cry-gal4(39)* (Klarsfeld et al., 2004). Females from each *gal4* line were crossed to males homozygous for either *10xUAS-IVSmyr::gfp* reporter inserted at *attP40* or *10xUAS-mCD8::gfp* reporter inserted at *attP2* (Pfeiffer et al., 2010). For the *GifM* template construction, we chose a *w;+;+* background that had been pre-isogenized to CantonS (wild-type). Janelia Farm lines and non-*gal4* lines were obtained from the Bloomington stock center.

#### 2.2.2. Adult brain immunolabeling

##### 2.2.2.1. Dissection

Four to six day-old male brains were dissected in phosphate-buffered saline (PBS).

##### 2.2.2.2. Fixing the tissue

The samples were transferred immediately after dissection into 4% paraformaldehyde (PFA) in PBS on ice and protected from light. After ensuring that the samples settled to the bottom of the well, the brains were placed at 4°C overnight or room temperature (RT) for 1 h.

##### 2.2.2.3. Washing and permeabilization

Samples were washed six times at RT with cold PAT (Jenett et al., 2012) on the rocking mixer for 10 min per wash and then in PAT with 1% Triton X-100 for 10 min, for tissue permeabilization.

##### 2.2.2.4. Blocking and primary antibody

Brains were then incubated in blocking buffer (1% bovine serum albumin in PAT) for 2 h at RT or overnight at 4°C after which the blocking buffer was replaced with either a mouse nc82 antibody concentrate (Developmental Studies Hybridoma Bank, Iowa City, IA) at 1:2,000 dilution in PAT for the template brain, or a mixture of two primary antibodies in PAT: nc82 and chicken anti-GFP (Invitrogen A10262) at respectively 1:2,000 and 1:1,000 dilution. For co-expression experiments we added a rabbit anti-PDF antibody at 1:10,000 dilution (Neosystem, custom made). The samples were incubated in the primary antibody for 48–72 h at 4°C in the dark. The nc82 antibody labels synapses and serves as a marker for neuropil. It is a mouse monoclonal antibody from a large library generated against *Drosophila* head homogenates (Wagh et al., 2006).

##### 2.2.2.5. Secondary antibody

After the primary antibody incubation, samples were returned to RT and were given six washes as previously described. After the last wash solution, brains were incubated with a secondary antibody solution. The secondary antibodies used were Alexa Fluor 488 goat anti-chicken (Invitrogen A11039), Alexa Fluor 647 goat anti-mouse (Invitrogen A21236) and FluoProbes 547H goat anti-rabbit (Interchim FP-CB1050), all diluted (1:1,000) in PAT and incubated for 48–72 h at 4°C in the dark. After the secondary antibody incubation, tissues were washed six times with PAT as in previous steps.

#### 2.2.3. Confocal imaging

Whole-mount brains were mounted on a 76 × 26 mm glass microscope slide (KnittelGlass) to which pairs of Paper Reinforcement Rings had been applied. The samples were mounted in Prolong Gold mounting solution (Invitrogen). Spacers were covered with a cover glass (KnittelGlass, #1 thickness, 0.17 mm) held in place by nail polish. Image acquisition was done sequentially on a Leica TCS SP8 upright microscope with a 25 × 0.95 NA plan-apochromat water immersion objective. The original image data consisted of 1, 024 × 1, 024 × ~200 voxels, with a voxel size of 0.60 × 0.60 × 0.98 μm. Images were acquired with a 12-bit dynamic range. A frame average of two successive scans was applied. Fluorescence emission from the 488, 547, and 647 nm was imaged using the 488, 561, and 633 nm lasers, respectively. The laser power was increased along the z-axis to compensate for signal attenuation.

### 2.3. Image registration and atlas generation

Group-wise intensity templates were constructed with the Advanced Normalization Tools (ANTs) software (Avants et al., 2011), using an algorithm building an average shaped brain that follows a two-step strategy. First, the intensity images of all brains used for building the atlas were aligned onto a randomly selected brain using rigid transforms (rotations and translations), which were determined by maximizing the mutual information (a criterion measuring the structural congruence between two images) using a multi-resolution iterative gradient descent algorithm. The aligned images were then voxel-wise averaged to create an initial, blurry average brain. Next, all individual brains were warped non-linearly on this average using symmetric diffeomorphic image registration (SyN) (Avants et al., 2008) with cross-correlation as similarity metric. A new average was calculated by combining the co-registered brains. The non-linear registration was refined by repeating this step at four resolution levels to converge to an optimal average template. This algorithm was run through the *buildtemplateparallel.sh* (Avants et al., 2010) ANTs script.

For combining the co-registered images into a final template image, we experimented with the default ANTs strategy (state-of-the-art method in MRI), which generates a normalized average image by voxel-wise averaging followed by sharpening with a Laplacian kernel. In addition, we implemented in Matlab an alternative strategy in which the template intensity image was generated by computing a voxel-wise median over the co-registered images, with no subsequent sharpening step.

The anatomical label image associated with the template was obtained by applying to each individual label image the diffeomorphic transformations computed from the corresponding reference (nc82) image, followed by a per-voxel majority voting over all warped label images. Labels were interpolated using the nearest neighbor method.

Individual (non atlas) brain images were registered against atlas templates using the *antsIntroduction.sh* ANTs script (Avants et al., 2010), which performs an initial rigid registration with mutual information as similarity metric followed by non-rigid registration with SyN and cross-correlation as similarity measure.

### 2.4. Evaluation metrics

Using test brains, each providing an intensity image and its associated label image, different atlases were evaluated for their performances regarding either segmentation or registration. In the segmentation task, the atlas labels were transformed into the coordinate system of each individual test brain to be compared with its labels. In the registration task, the labels of each individual test brain were mapped into the atlas coordinate system to be compared with the labels of other test brains.

Region-to-region matching in individual or atlas coordinate system was quantified using the Dice coefficient. For any region *R*_*i*_, the Dice coefficient provides a normalized measure of the overlap between two instances $R_{i}^{A}$ and $R_{i}^{B}$ that have been transformed into a common space by the registration procedure. The Dice coefficient is defined as

(1) $$\begin{array}{r} \text{Dice}\left(R_{i}\right)=2\frac{\left|R_{i}^{A}\cap R_{i}^{B}\right|}{\left|R_{i}^{A}|+|R_{i}^{B}\right|} \end{array}$$

where |·| denotes the size (number of voxels) of a region.

The average boundary error, expressed in absolute distance units, was computed as the mean symmetric Euclidean distance. For region *R*_*i*_, we computed the mean Euclidean distance $d_{i}^{A,B}$ between each boundary point on $R_{i}^{A}$ and the closest point on $R_{i}^{B}$. The symmetric computation was performed to obtain $d_{i}^{B,A}$. The symmetric Euclidean distance for region *R*_*i*_ was then defined as

(2) $$\begin{array}{r} \text{Symmetric Euclidean distance}\left(R_{i}\right)=\frac{d_{i}^{A,B}+d_{i}^{B,A}}{2} \end{array}$$

The maximum boundary error, also expressed in absolute distance units, was computed as the mean symmetric Hausdorff distance. For region *R*_*i*_, the Hausdorff distance $h_{i}^{A,B}$ was computed as the maximum distance between any boundary point on $R_{i}^{A}$ and its closest neighbor on $R_{i}^{B}$. The symmetric computation yielded $h_{i}^{B,A}$, and the symmetric Hausdorff distance was obtained as

(3) $$\begin{array}{r} \text{Symmetric Hausdorff distance}\left(R_{i}\right)=\frac{h_{i}^{A,B}+h_{i}^{B,A}}{2} \end{array}$$

The mean symmetric Euclidean distance was also used to quantify residual distances between axonal traces in the template space. The above definition was applied, replacing anatomical regions with the 3D skeletons of individual traces. In addition, the obtained distances were divided by the equivalent spherical radius of the templates. This normalization was applied to compensate for size variations between templates.

### 2.5. 3D image database browsing and querying

#### 2.5.1. Tefor BrainBase: database of registered images

As a performance test of our registration algorithm, we registered the published data of the Janelia Farm Gal4 collection (http://flweb.janelia.org/cgi-bin/flew.cgi, Jenett et al. 2012) into the coordinate system of our inter-sex template. Over 3,350 3D images of expression patterns were processed and their metadata imported into PostgreSQL database using the BrainBase storage framework (http://braingazer.org).

#### 2.5.2. BrainBaseWeb 2.0: lightweight database webinterface

The BrainBaseWeb interface of VRVis (http://vrvis.at) was widely redesigned and functionally enhanced to access the registered images stored in the Tefor database. BrainBaseWeb is the primary user interface to efficiently browse and retrieve confocal microscopy data and related metadata like annotated anatomical structures of imaging and registration parameters from the BrainBase (http://braingazer.org) storage framework. It provides the user with reactive client side slice viewers as well as 3D visualization of the expression patterns in a standard web client as Chrome, Firefox or Safari. Employing intelligent caching techniques, data are loaded and temporarily stored on demand only, which accelerates data access and visualization. Besides a traditional semantic search engine, BrainBaseWeb 2.0 presents as a new feature the spatial query, which previously was only available in the desktop application BrainGazer (see below): using a brush-tool, complex queries for expression patterns can be submitted without any prior knowledge.

#### 2.5.3. BrainGazer: desktop application with enhanced functionality

Even more complex, annotation based queries on the BrainBase can be constructed with VRVis' BrainGazer desktop application for Windows or OSX (Bruckner et al., 2009). In addition to the above described spatial query, BrainGazer provides tools for combinatorial semantic and non-semantic queries, which are constructed through intuitive graphical user interfaces. In contrast to BrainBaseWeb, BrainGazer is installed on the local computer and has full access to the graphics hardware of the local system. This allows for high-end 3D rendering of high resolution data, which can be directly downloaded from the data pool or results page of a query. On modern hardware this point makes the BrainGazer a powerful tool for in-detail analysis of atlas data.

## 3. Results

### 3.1. Group-wise atlases outperformed individual atlases

At least four of the most popular adult *Drosophila* templates are brains of single individuals (Rein et al., 2002; Jenett et al., 2006, 2012; Chiang et al., 2011). The brains in individual templates are frequently chosen for their apparent or objective representativeness of a whole population of specimens. Here, we evaluated quantitatively to what extent the arbitrary selection of a reference brain potentially affects the performance of the template in registration and segmentation tasks. In addition, we also evaluated how using group-wise templates built by pooling several individuals may improve these performances.

**Figure 1:**
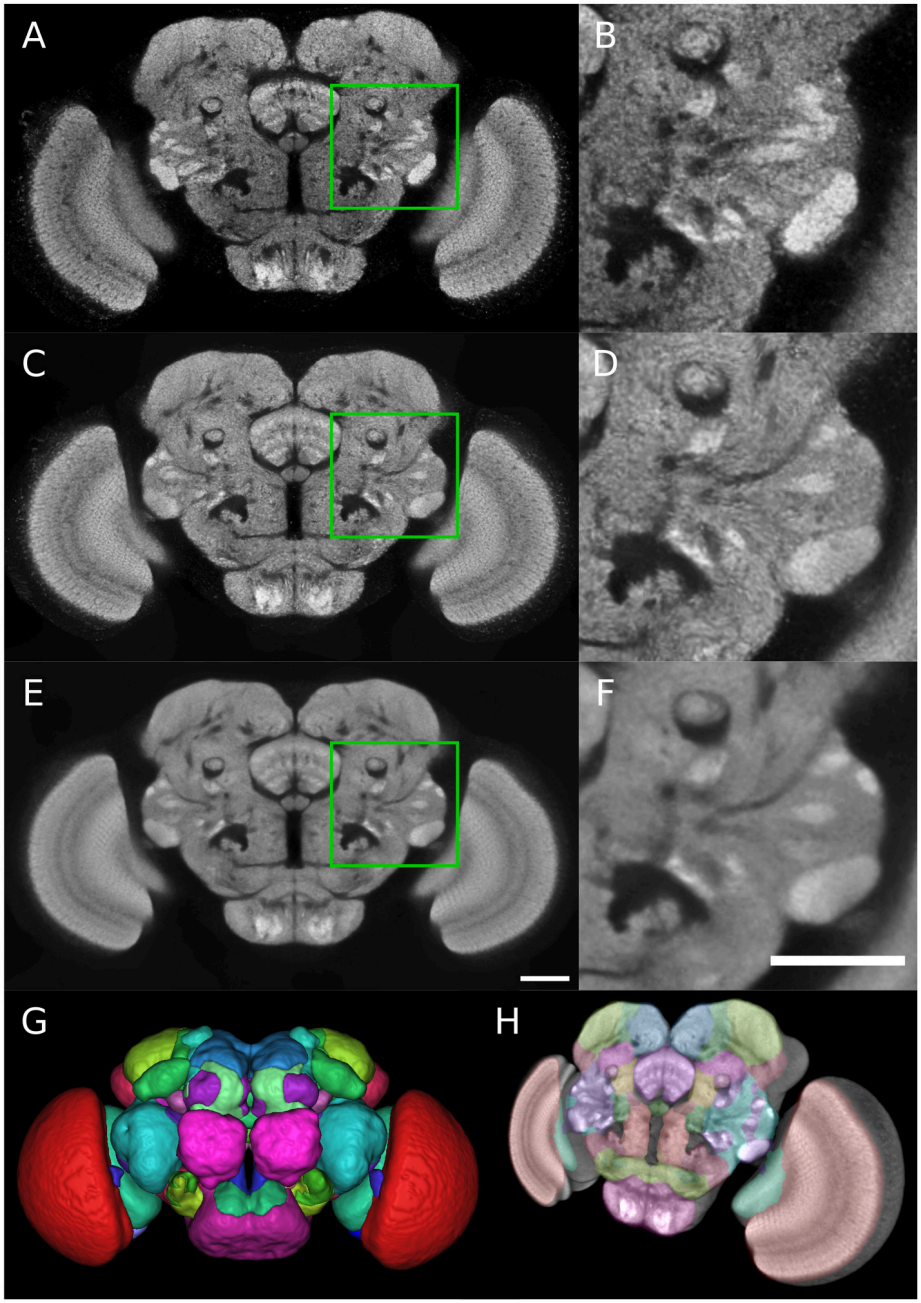
Group-wise inter-sex atlas of *Drosophila* adult brain. **(A)** Central slice of individual male brain used in the construction of the template. **(B)** Magnified view of the green square in **(A)**. **(C)** Central slice of inter-sex template created using the sharpened normalized average (default ANTs method). **(D)** Magnified view of the green square in **(C)**. **(E,F)** Same as **(C,D)** for the inter-sex template created using the median. Scale bar: 50 μm. **(G)** 3D surface rendering of anatomical regions of the Ito et al. (2014) atlas following its registration on the median template (color scheme according to Ito et al., 2014). **(H)** 3D view of central template slice with overlay of Ito et al. (2014) atlas regions.

We exploited the Würzburg dataset (see section 2.1), composed of male and female brain images containing each a nc82 intensity channel (reference channel; Figures 1A,B) and the corresponding anatomical region labels delineated by human experts (Rein et al., 2002; Jenett et al., 2006). The same experiment was separately performed for females and for males. Ten specimens (the *template* set) were used both as individual templates and for building group-wise templates. Twelve distinct samples (the *test* set) were used for evaluating these templates. To prevent biasing the evaluation process by size effects, the *template* and *test* sets were created so they presented comparable distributions of brain volumes.

Using the *template* set, two group-wise atlases were built. The same registration procedure, combining linear then non-linear (diffeomorphic) transforms, was applied to co-register the intensity images in the set. The two atlases differed by their template intensity images, which were either a sharpened average image computed according to the default ANTs method (*Mean* template) or a median image (*Median* template). The two atlases shared the same anatomical label image, which was calculated by applying majority voting to the co-registered individual label images. The 12 test brains were independently registered to each of the 10+2 individual and group-wise templates. The test brains were registered on these templates using the same diffeomorphic approach as used for generating the group-wise templates.

**Figure 2:**
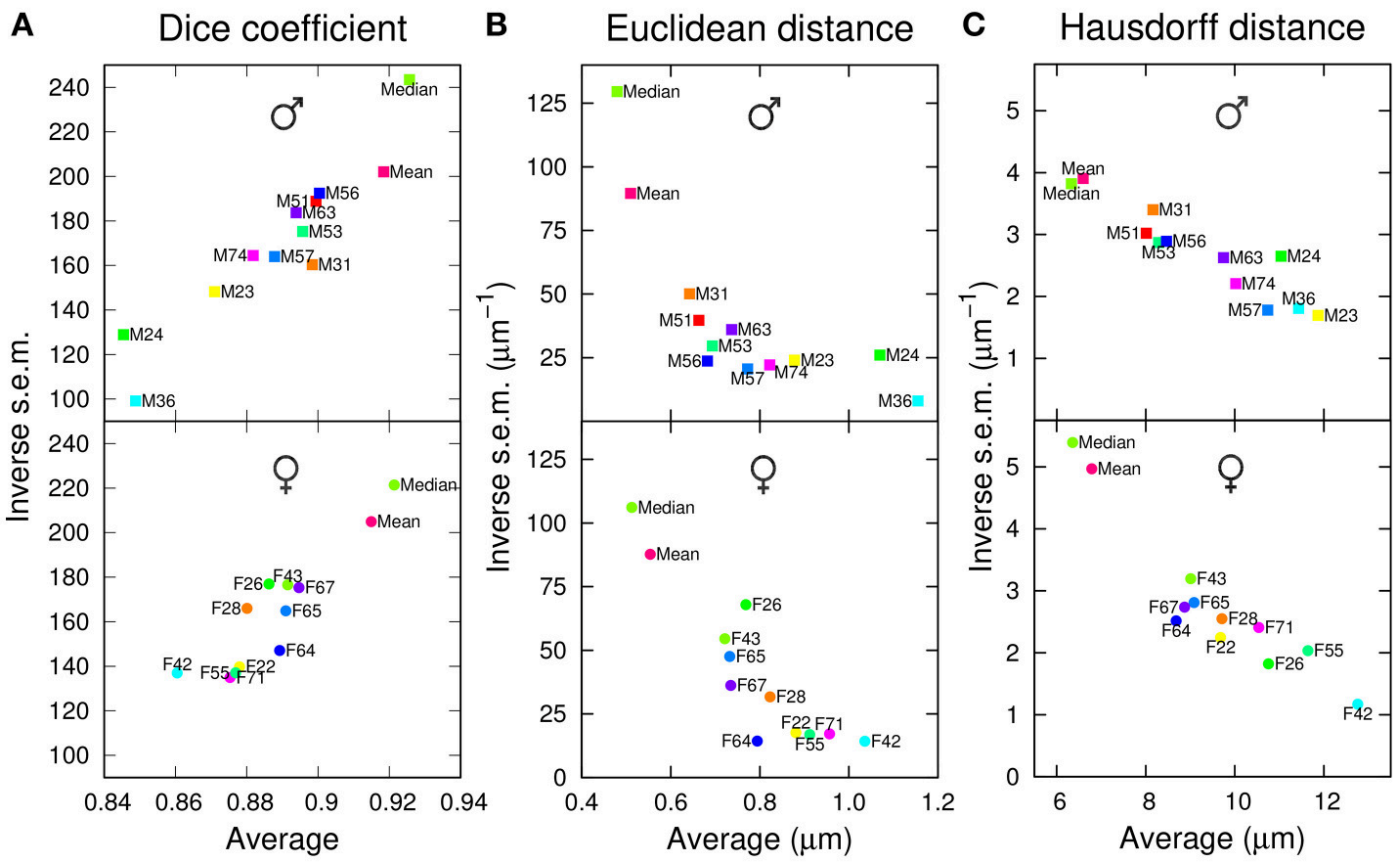
Segmentation performances of individual and group-wise templates. The graphs plot, for males (Top) and females (Bottom), the inverse standard error of the mean (s.e.m.) as a function of the mean of each segmentation metric, computed in the original space of test brains and over all anatomical regions. **(A)** Dice coefficient. **(B)** Average inter-surface Euclidean distance. **(C)** Maximum (symmetric Hausdorff) inter-surface distance. The single male and female brains used as individual templates are labeled as F*N* or M*N*. For each gender, *Mean* and *Median* design the mean and median intensity group-wise templates, respectively.

To evaluate the performance of the 10+2 templates in the segmentation task, the anatomical labels of each template were transformed into the coordinate system of each individual brain of the *test* set. The agreement between template and test brain labels was first evaluated using the Dice coefficient (the higher this coefficient, the better the overlap between specimen and atlas labels). We observed that the two group-wise atlases systematically exhibited higher and less variable Dice coefficient values compared with individual ones (Figure 2A). In addition, computing a median intensity template increased the precision and accuracy of the segmentation process compared with the standard ANTs averaging procedure. All these observations were made for both males and females (Figure 2A).

To quantify the average absolute amplitude of misalignment, we measured the Euclidean surface distance between label regions from atlases and test brains (the smaller this measure, the better the matching). On average, the border to border distance was below voxel size (voxel diagonal = 1.4 μm), suggesting sub-voxel accuracy (Figure 2B). However, the group-wise templates exhibited smaller and less variable residual distances between registered regions, and the *Median* template showed better performance compared with the ANTs average one (*Mean* template).

Since large deviations may be smoothed out by computing a mean Euclidean distance, we next examined the average maximum border to border distance, as quantified by the symmetric Hausdorff distance. We obtained that the maximum error between registered label regions could, on average, span the equivalent of about 10 voxels (Figure 2C). Again, the group-wise templates outperformed the individual ones. Overall, with both mean and maximum residual distances, the group-wise atlases yielded about 40% reduction in segmentation error compared with individual ones.

For males as well as for females, we observed that the individual atlases ranked differently depending on the considered evaluation criterion. For example, M24 performed the worst, second worst, and third worst, according to Dice coefficient, average Euclidean distance, and Hausdorff distance, respectively (Figure 2). Conversely, the group-wise *Mean* and *Median* atlases consistently ranked second and first with the three criteria (Figure 2). Overall, we concluded that group-wise templates outperformed individual ones, by increasing both the precision and accuracy of registration-based automatic segmentation, and that computing a median rather than an average intensity image further improved the performances of the group-wise template.

**Figure 3:**
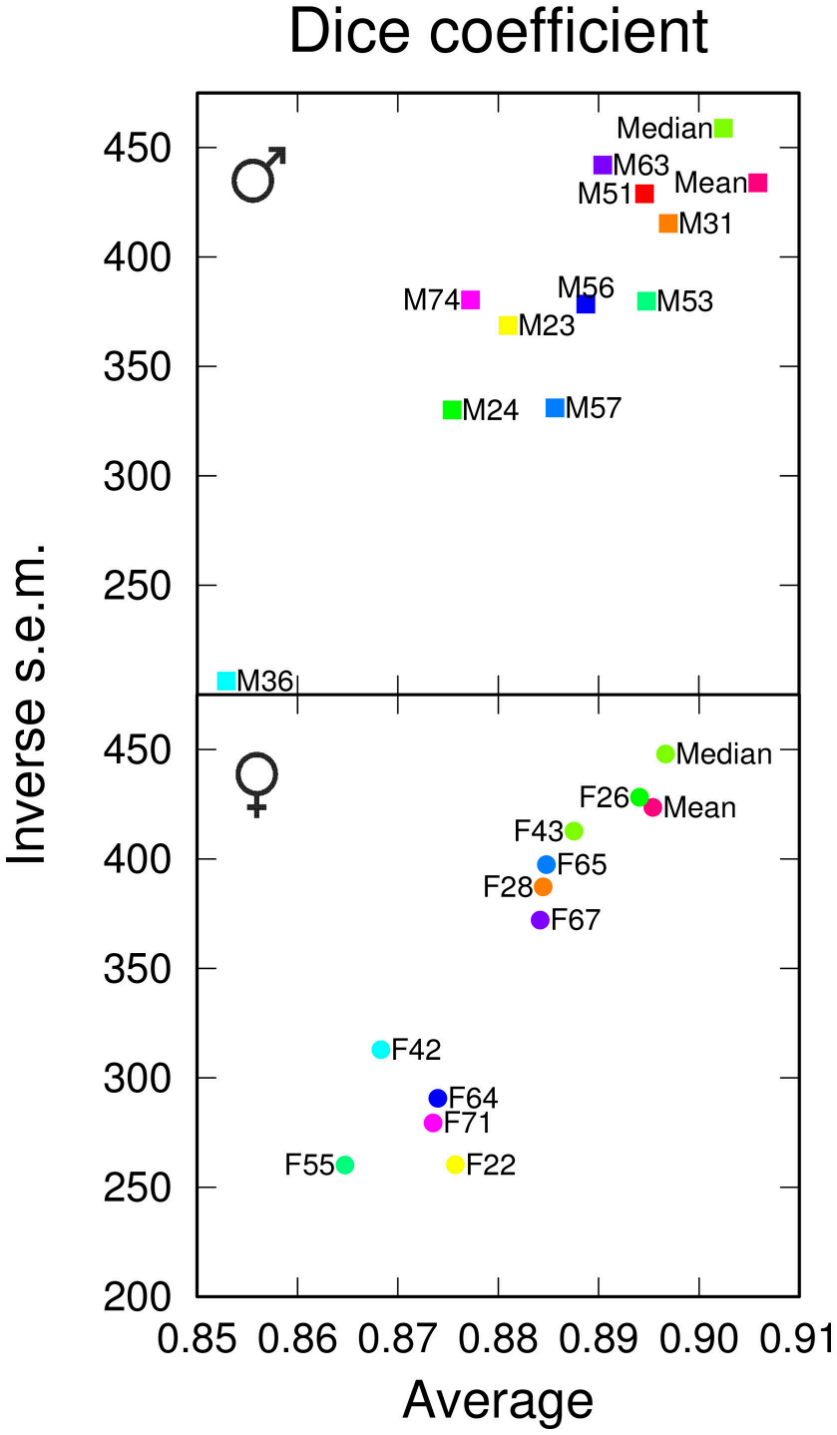
Registration performances of individual and group-wise templates. The graphs plot, for males **(Top)** and females **(Bottom)**, the inverse standard error of the mean (s.e.m.) as a function of the mean of the Dice coefficient, computed in the template space and over all anatomical regions. The single male and female brains used as individual templates are labeled as F*N* or M*N*. For each gender, *Mean* and *Median* design the mean and median intensity group-wise templates, respectively.

We next examined the performances of the different templates in the registration task. Following registration on each of the 12 templates, pairwise comparisons between homologous regions from different individuals were performed. Since absolute distance measurements were potentially affected by size differences between templates, we only measured the Dice coefficient. In males, the *Median* template exhibited a slightly smaller overlap value than the *Mean* template (Figure 3). In females, however, the *Median* template performed better than the *Mean* one, and for both genders, the *Median* template exhibited less variability in the registration error (Figure 3). There were large fluctuations between individual templates and, though the difference was relatively smaller when compared to segmentation results (Figure 2A), the group-wise templates yielded higher Dice coefficient values compared with individual ones (Figure 3). Hence, in the registration task, group-wise templates yielded increased precision and accuracy as observed for the segmentation task.

### 3.2. Inter-sex atlas compared similarly to sex-specific atlases

Given the known sex dimorphism in *Drosophila* (Cachero et al., 2010; Yu et al., 2010; Ren et al., 2016), female-specific and male-specific templates are very common in the fruit fly community (Rein et al., 2002; Chiang et al., 2011; Jenett et al., 2012). However, inter-sex templates created by combining female and male individuals also exist (Jefferis et al., 2007; Cachero et al., 2010; Yu et al., 2010; Costa et al., 2016). We thus asked whether inter-sex atlases could be used in place of sex-specific atlases. We addressed this question using five female brains and five male brains as individual atlases and as components of two inter-sex group-wise *Mean* and *Median* atlases (with average- and median-intensity templates, respectively).

The two obtained group-wise templates retained the crisp local contrast associated with neuropil structures visible on individual images (Figures 1A–F). However, the *Median* template exhibited a better signal-to-noise ratio than the *Mean* one (compare Figures 1E,F to Figures 1C,D). Indeed, the template image obtained with the median operator was smoother compared to the sharpened mean, with a comparable global contrast. The higher intensity heterogeneity in the mean-based template likely resulted from the sharpening step coupled to the averaging procedure in the ANTs toolkit.

**Figure 4:**
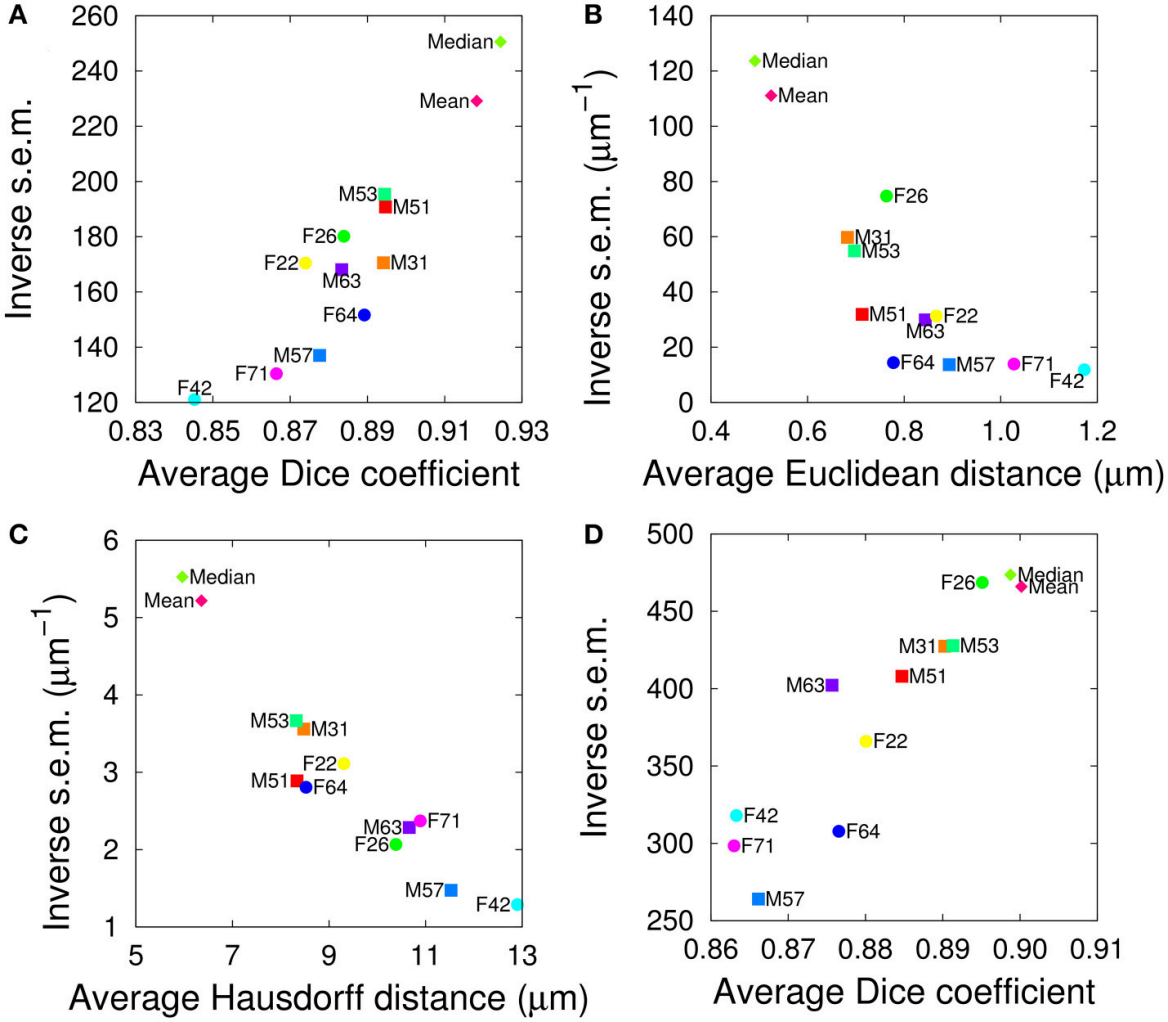
Evaluation of inter-sex templates in segmentation **(A–C)** and registration **(D)** tasks. The graphs plot the inverse standard error of the mean (s.e.m.) as a function of the mean of each segmentation metric, computed over all anatomical regions either in the test brain spaces **(A–C)** or in the template spaces **(D)**. **(A,D)** Dice coefficient. **(B)** Average Euclidean inter-surface distance. **(C)** Maximum (symmetric Hausdorff) inter-surface distance. The single male and female brains used as individual templates are labeled as F*N* or M*N*. *Mean* and *Median* are the mean and median intensity group-wise inter-sex templates, respectively.

We evaluated the segmentation and registration performances of the 10+2 atlases using a test set of twelve additional samples, containing six female and six male brains. For both tasks and for all evaluation criteria, the range of variations between individual templates was comparable to the one observed in the single sex cases, suggesting the absence of impact of gender on the performance of the templates (Figure 4). In addition, the group-wise atlases again outperformed the individual ones, and the median-intensity template was globally superior to the average-intensity one.

**Figure 5:**
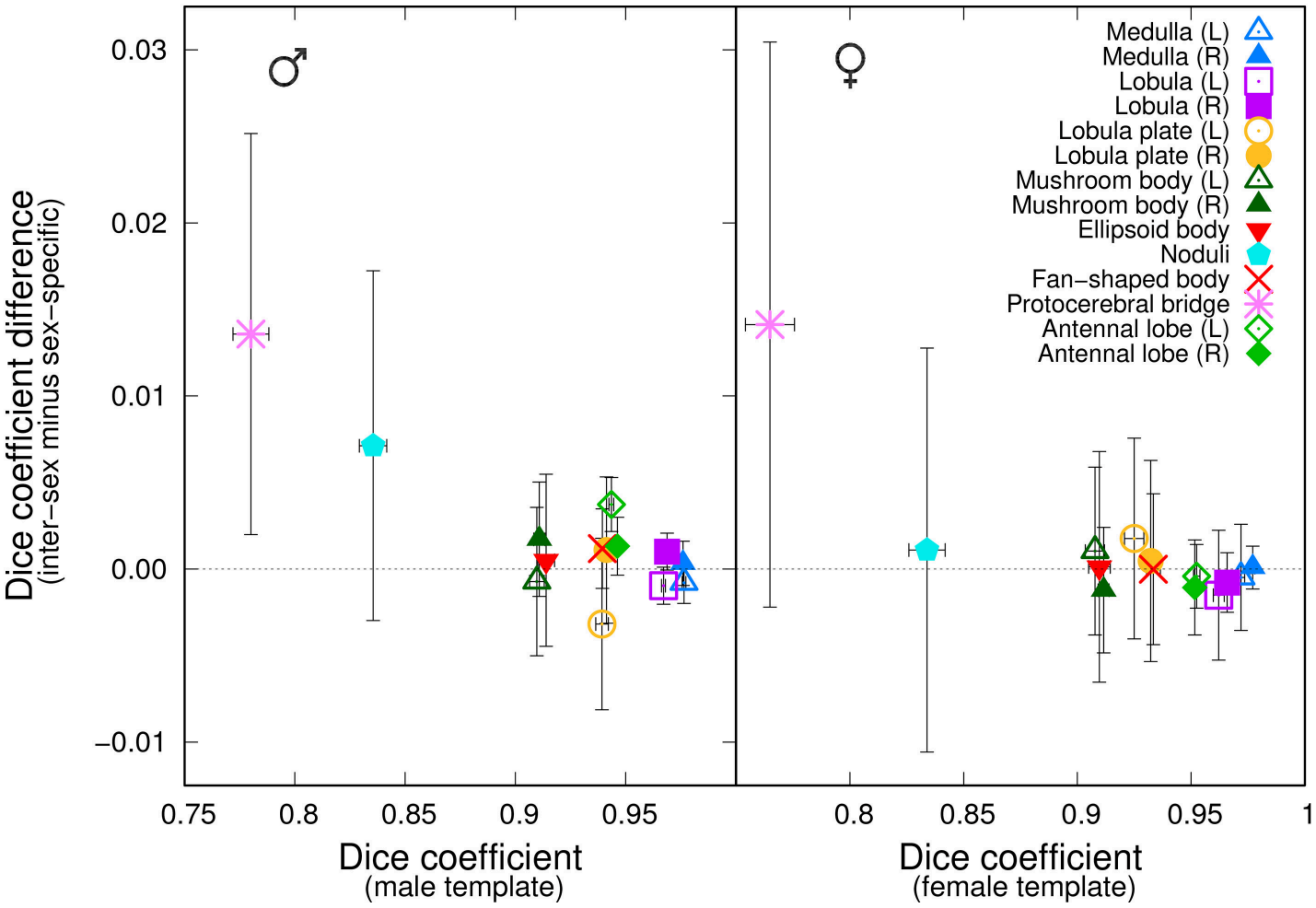
Comparison of the performances of inter-sex and sex-specific templates in the segmentation task. The graphs plot, for each of the 14 anatomical regions, the difference between the average Dice coefficient computed with either the inter-sex or the sex-specific templates as a function of the Dice coefficient computed with the sex-specific template. **(Left)** Males; **(Right)** Females. Error-bars: s.e.m.

We next asked whether a group-wise inter-sex template compared similarly to a group-wise sex-specific template when processing individuals of that sex. We independently registered twelve male brains against the *Median* group-wise inter-sex template. We compared the results with those obtained by registering the same twelve brains against the *Median* group-wise male-specific template. An analogous experiment was performed using female samples. For each sex and for each anatomical region, we computed the difference between the Dice coefficient in the test brain coordinate systems when using either the inter-sex or the sex-specific atlases. We plotted this difference as a function of the Dice coefficient obtained using the sex-specific template (Figure 5). For most anatomical regions, the average difference was close to zero, suggesting that the inter-sex atlas compared similarly to the sex-specific ones. The protocerebral bridge was a noticeable exception. Surprisingly, the inter-sex template exhibited better performance than the two sex-specific ones for the automatic segmentation of this structure in the test brain coordinate frame. A similar, though less pronounced, effect was also observed for the noduli in the male group.

For each sex, we objectively compared the distributions of the Dice coefficient obtained with the inter-sex and sex-specific atlases. In the female group, there was no significant difference between female-specific and inter-sex distributions (Wilcoxon signed rank test, *P* = 0.49). In the male group, however, this difference was significant (*P* < 0.01). This could be attributed mainly to the results obtained for the protocerebral bridge, since excluding this region from the test abolished the difference (*P* = 0.18). Given the low average Dice coefficient values obtained with sex-specific atlases for the protocerebral bridge and the difficulty to accurately manually delineate this narrow and elongated structure, we concluded that overall the median-intensity group-wise inter-sex atlas performed similarly to sex-specific atlases.

### 3.3. The group-wise atlas converged with a few individuals

The results reported above show that group-wise atlases yield better performances in the segmentation and registration tasks than individual ones. However, the performances of group-wise templates likely depend on the number of individuals used to establish these templates. We thus asked whether the number of individuals in our templates (set to ten above) was optimal or not. We generated ten series of inter-sex atlases, each series containing an increasing number of individual brains with a final maximum value of twelve individuals. Female and male specimens were successively introduced in a random order, alternating between sexes. The first individual in five series was a female, and was a male in the other five.

The obtained incremental atlases were used to automatically segment and register an independent set of twelve manually segmented brains (six female and six male). Segmentation and registration performances were evaluated using the Dice coefficient measured in the individual or the atlas space, respectively.

**Figure 6:**
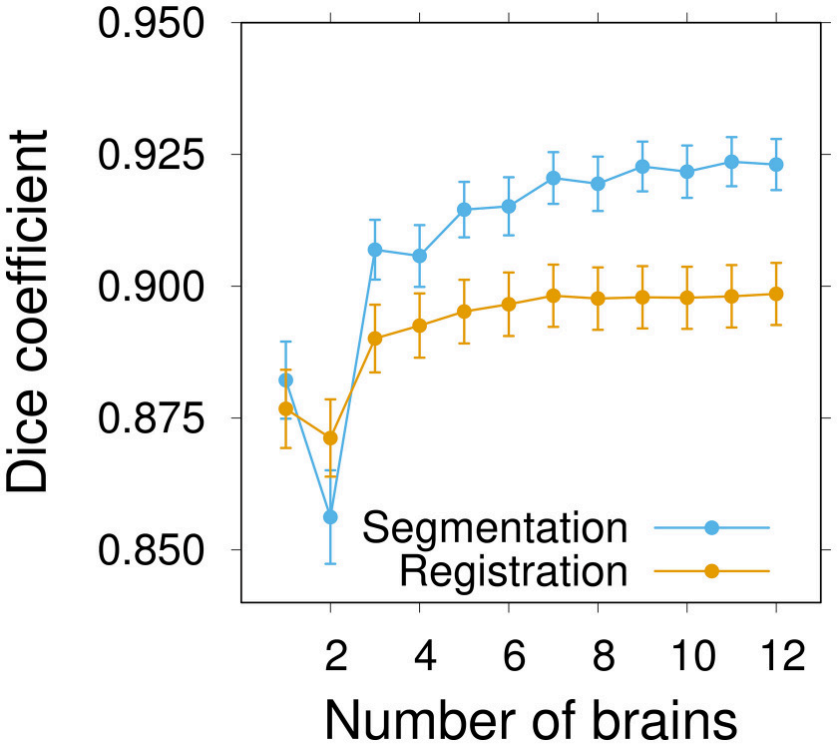
Influence of the number of brains on inter-sex atlas performance in segmentation and registration tasks. Group-wise inter-sex atlases were created with an increasing number of brains. Performance was quantified by computing the average Dice coefficient of segmented anatomical regions of male and female test brains after segmentation (*blue*) or registration (*orange*). Performance measures were averaged over 10 repeats. Error bars: s.e.m.

The average segmentation performance globally increased with the number of individuals and converged to a plateau around *n* ≃ 9–10 individuals (Figure 6). The average registration performance exhibited a similar pattern. However, convergence was reached sooner than for segmentation, around *n* ≃ 7 brains. In addition, the performance level reached upon convergence was higher for segmentation than for registration. This was a probable consequence of the fact that the label images of group-wise atlases (which are only used in the segmentation task) are smoother than individual ones. For segmentation as for registration, there was a pronounced increase in performance at the transition between 2 and 3 brains per atlas, thus further emphasizing the benefits of statistical atlases. It is likely that the poor performance of some of the individual atlas brains is smoothed out in statistical atlases as soon as they are in a minority, which generally happens as soon as there are two other individuals. Overall, the results of this experiment strongly suggested that convergence had been reached in the inter-sex group-wise atlas after the integration of about ten individuals.

### 3.4. Comparison with publicly available templates

After demonstrating the superiority of our group-wise strategy with respect to individual brain templates, the next step was to evaluate its performance against publicly available whole-brain templates. We tested our median-intensity group-wise inter-sex template against five alternatives: the FlyLight template (*JFRC2010*, single female brain, stained with nc82) (Jenett et al., 2012), an average inter-sex template from the FlyCircuit database (*FCWB*, Chiang et al. 2011) constructed by Gregory Jefferis's lab (Dlg staining) (Ostrovsky and Jefferis, 2014) and the three new average, artificially-symmetric templates from this same lab (*DmelF*, female-specific; *DmelM*, male-specific; *DmelIS*, inter-sex; all three with nc82 staining) (Ostrovsky et al., 2014).

Since not all public templates share the same anatomical labels (some do not have labels at all), the comparison was done for the registration task only. Twelve anatomically annotated test brains (six females and six males) were registered and warped into the coordinate system of each evaluated template. The Dice coefficient was averaged over all structures and pairs of registered test brains. As above, the two distance metrics were not used in this evaluation because of size differences across templates. Since the evaluated set of templates contained both sex-specific and inter-sex templates, we performed three evaluations using female only, male only, and both female and male test brains.

**Figure 7:**
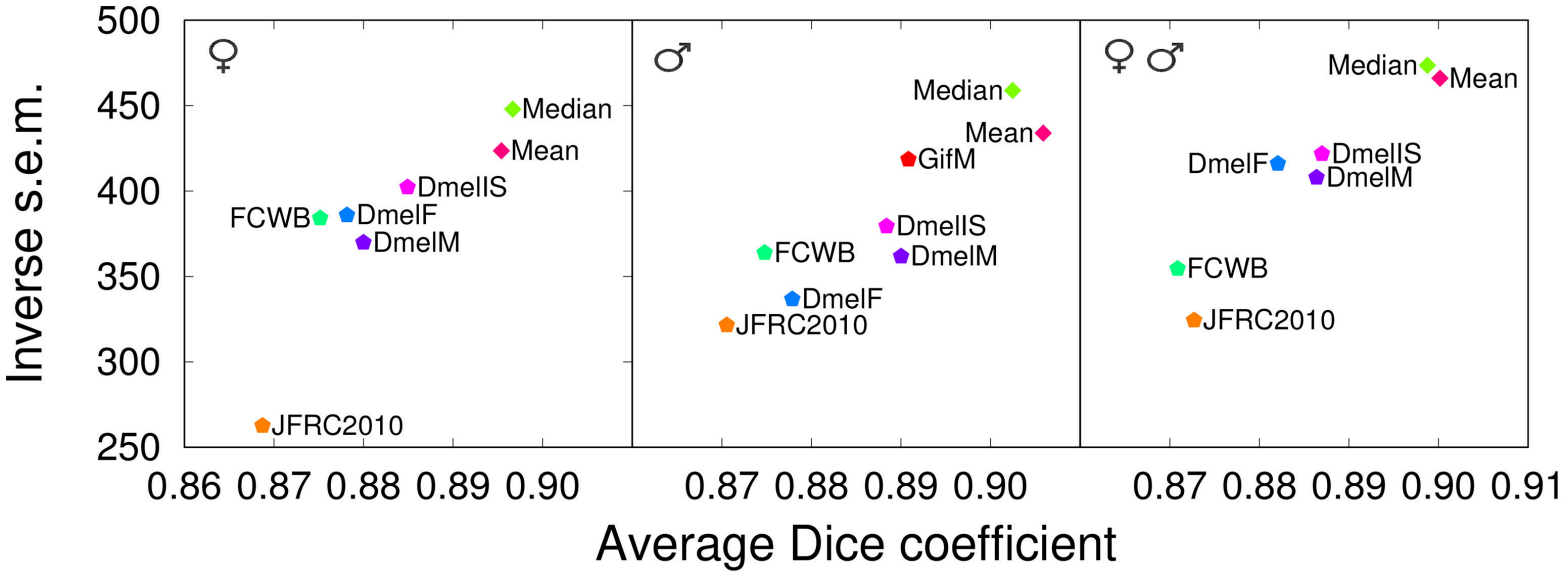
Comparison with other templates (registration task). Dice coefficient obtained with our proposed group-wise templates (*Median* and *Mean*) and with other publicly available templates evaluated by registering female brains **(Left)**, male brains **(Middle)**, or both female and male brains (evaluation in template space). The male test set **(Middle)** was also used to evaluate the performance of an additional male-specific template (*GifM*) built using newly acquired nc82-stained samples. *JFRC2010*: single female template from the FlyLight database; *FCWB*: an inter-sex template that combines female and male brains from the FlyCircuit database; *DmelF, DmelM*, and *DmelIS*: symmetric group-wise female-specific, male-specific, and inter-sex templates, respectively, from Jefferis' lab.

The results show that our templates yielded the highest mean Dice coefficient between registered anatomical labels in template space for the three evaluation cases (Figure 7). Interestingly, the only single-individual template involved in this comparison (*JFRC2010*) had the lowest scores, providing additional, independent support to the conclusion that group-wise templates are superior. The *FCWB* template had lower performance compared with the three nc82-based average templates from Jefferis' lab. As the *FCWB* was built using more individual brains (26) than the two sex-specific *DmelM* an *DmelF* templates (18 and 14, respectively), we interpreted this difference in performance as a possible consequence of having different staining between the template and the test brains. We noted that, despite they rely on larger numbers of individuals, the three *DmelM, DmelF*, and *DmelIS* templates tended to exhibit lower Dice scores compared with our group-wise templates. Since the intensity contrast was derived from the same nc82 staining in these templates, this suggested an improved performance due to the symmetric diffeomorphic registration algorithm used to generate our templates.

### 3.5. Robustness to image acquisition conditions

All evaluations of our group-wise atlases reported above have been against test brains belonging to the same “Würzburg” dataset, thus raising two questions. First, can the success of our strategy compared with alternative templates be explained by the common origin of template and test brains? Second, what is the robustness of our strategy to changes in the image acquisition conditions? To examine these issues, we constructed a new template (*GifM*) using ten male nc82-stained samples acquired in this study (the “Gif” dataset) and checked its performance by co-registering independent “Würzburg” male individuals. We used the same twelve male brains that were used as a test set in the above comparison with other templates.

The results obtained by evaluating registration using the Dice coefficient showed a partial sensitivity of template performance to image acquisition conditions (Figure 7, Middle). Indeed, the *GifM* template yielded lower precision and accuracy compared with our *Mean* and *Median* templates. However, the *GifM* template was still superior to the alternative templates. This suggested that differences in image characteristics alone could not completely explain the better performances of our strategy over the alternatives.

### 3.6. Registration of gene expression patterns

**Figure 8:**
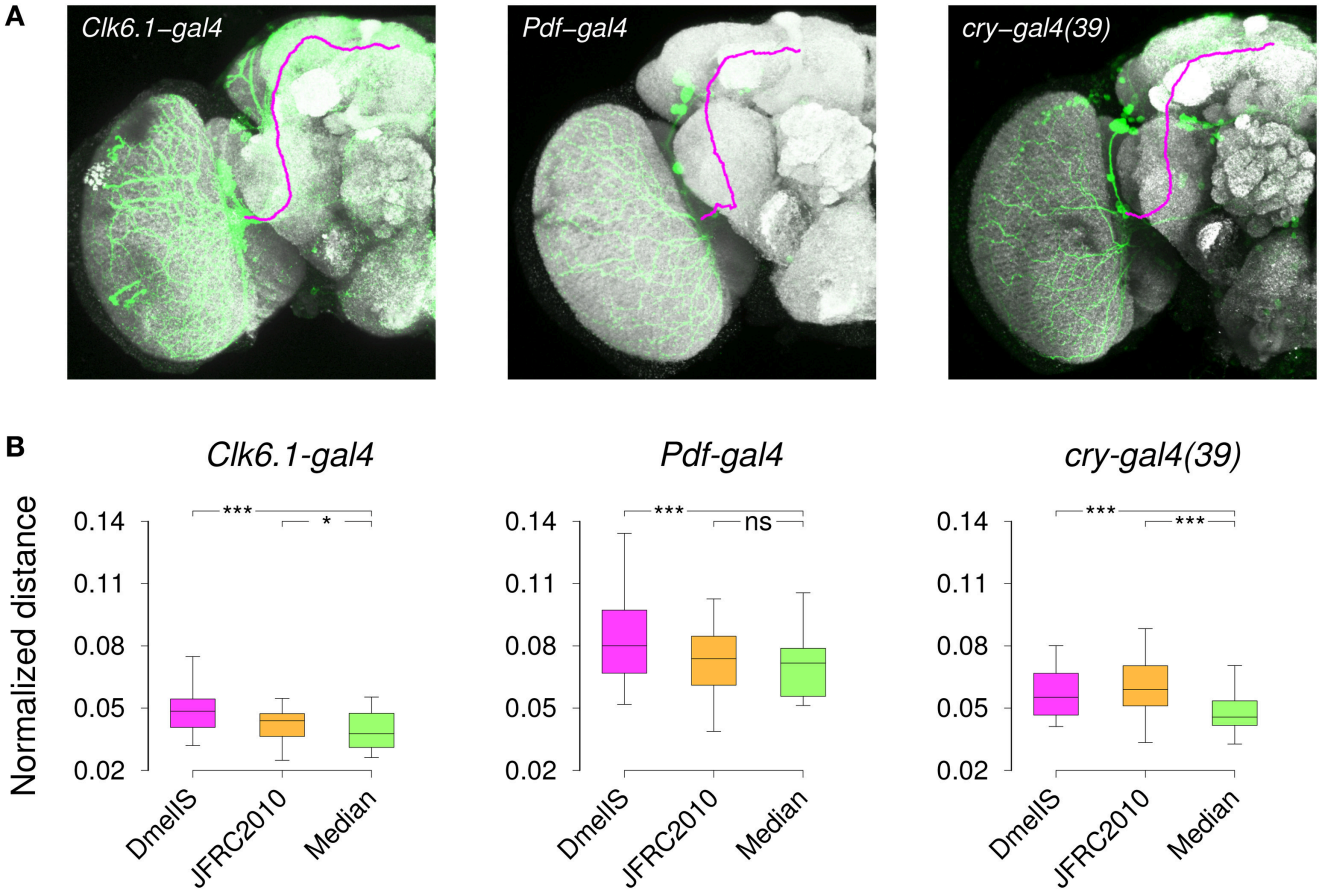
Registration of gene expression patterns. **(A)** Z-projections of individual 3D image stacks showing nc82-staining (*Gray*), transgene expression pattern (*Green*), and manually delineated 3D axonal traces (*Magenta*). **(B)** Distribution of the normalized distances between axonal projections after registering nc82-stained sample brains from *Clk6.1-gal4, Pdf-gal4*, or *cry-gal4(39)* transgenic lines. Registration was performed using either the FlyLight template (*JFRC2010*), the Jefferis' lab symmetric inter-sex template (*DmelIS*), or our median-intensity inter-sex template (*Median*). The results of the statistical comparison (paired Wilcoxon test) between the *JFRC2010* or *DmelIS* templates with the *Median* template are indicated as: ns, *P* > 0.05, *, *P* < 0.01, and ^***^, *P* < 0.001.

As an independent way of evaluating the templates, we compared gene expression patterns between different individuals of a same transgenic line following their registration into the template coordinate frame. We acquired brain images from several male specimens of three different transgenic lines (*Clk6.1-gal4, cry-gal4(39)*, and *Pdf-gal4*) that target overlapping subsets of clock neurons in the adult brain (Beckwith and Ceriani, 2015) and manually segmented labeled axonal projections on all of them (Figure 8A). Since the same neurons were labeled within a given transgenic line, the best template should be the one minimizing the residual distances between axonal traces, once individual images had been registered into the template space. Hence, following brain registration and trace skeletonization, we computed for each template an average point-to-point distance across all pairs of axonal traces. To prevent a template size effect, we normalized the measured absolute distances by the equivalent spherical radius of the template. We used three templates in this experiment, comparing our *Median* inter-sex template to the individual *JFRC2010* FlyLight template (Jenett et al., 2012) and to the group-wise inter-sex *DmelIS* template (Ostrovsky et al., 2014).

The normalized residual distances were variable from one line to the other, and the variability between lines was larger than the variability between templates. However, the median-intensity template produced lower residual inter-trace distances than the other two templates (Figure 8B). Except for the *Pdf-gal4* line, for which there was no difference between our *Median* and the *JFRC2010* templates, applying the Wilcoxon paired test confirmed the statistical significance of these differences. We thus concluded that the median-intensity inter-sex group-wise template was producing better trace registration compared with the alternatives.

### 3.7. Biological validation of registration-based predictions

As a performance test of our registration algorithm, we registered over 3350 3D images from the published Janelia Farm Gal4 collection (http://flweb.janelia.org/cgi-bin/flew.cgi, Jenett et al. 2012) into the coordinate system of our *Median* inter-sex template. The registered images and their associated metadata were imported into a database (the Tefor database, publicly accessible at http://fruitfly.tefor.net). In addition, we registered the anatomical labels of the Ito et al. (2014) atlas onto our template (Figures 1G,H; Supplementary Movie 1) and integrated them into the database, thus providing a comprehensive anatomical annotation of our statistical template. Therefore, the Tefor database is able to support a wide variety of atlas-based queries.

**Figure 9:**
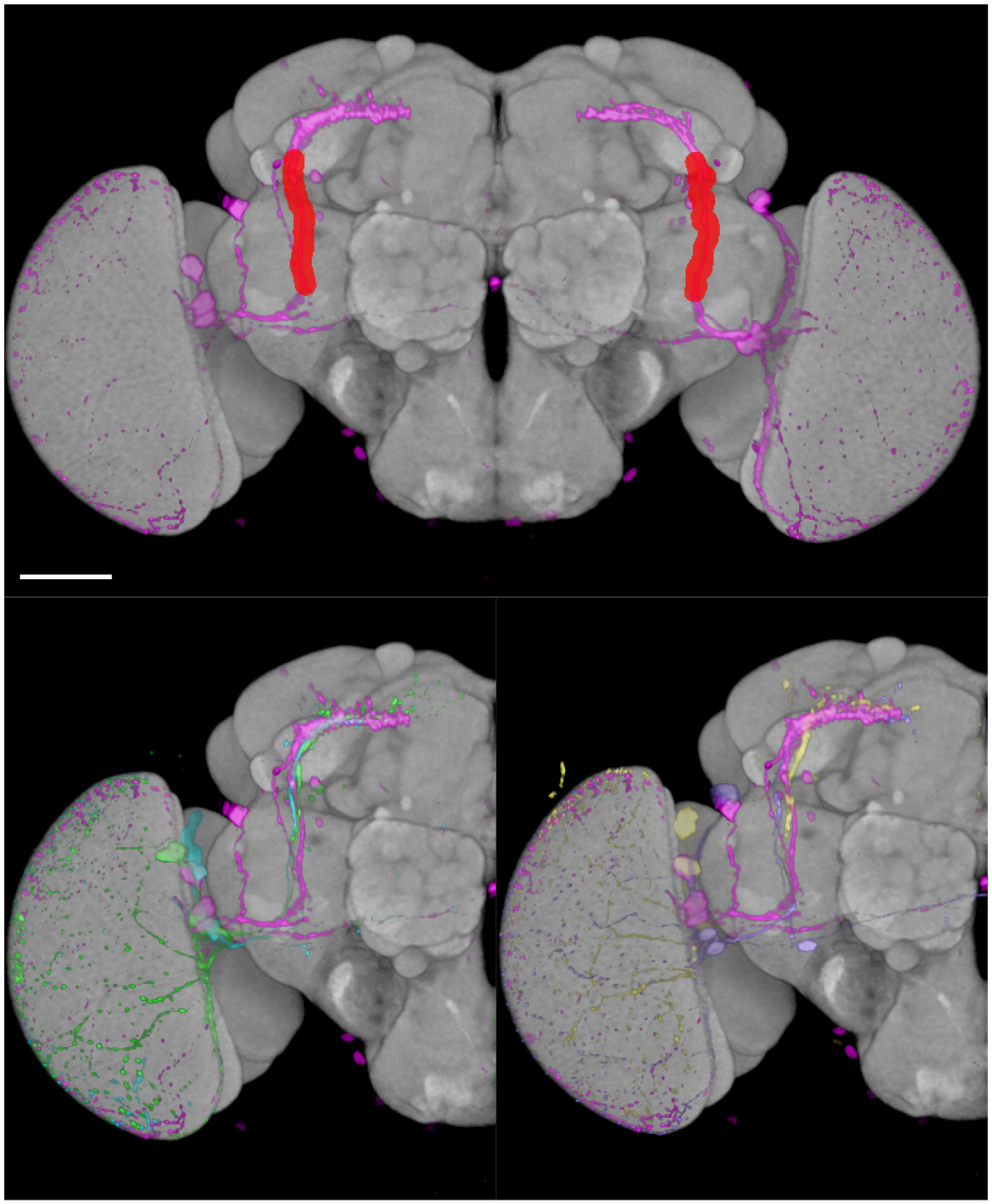
BrainGazer 3D space query over axonal tracts of five individual PDF-expressing sLNv profiles registered on the standard brain. **(Top)** The 3D space query brush tool is drawn (*red*) over one PDF profile (*pink*) in both brain hemispheres. **(Bottom)** The same PDF profile is shown with the four other PDF profiles on one hemisphere to illustrate individual variability. Scale bar: 50 μm.

**Figure 10:**
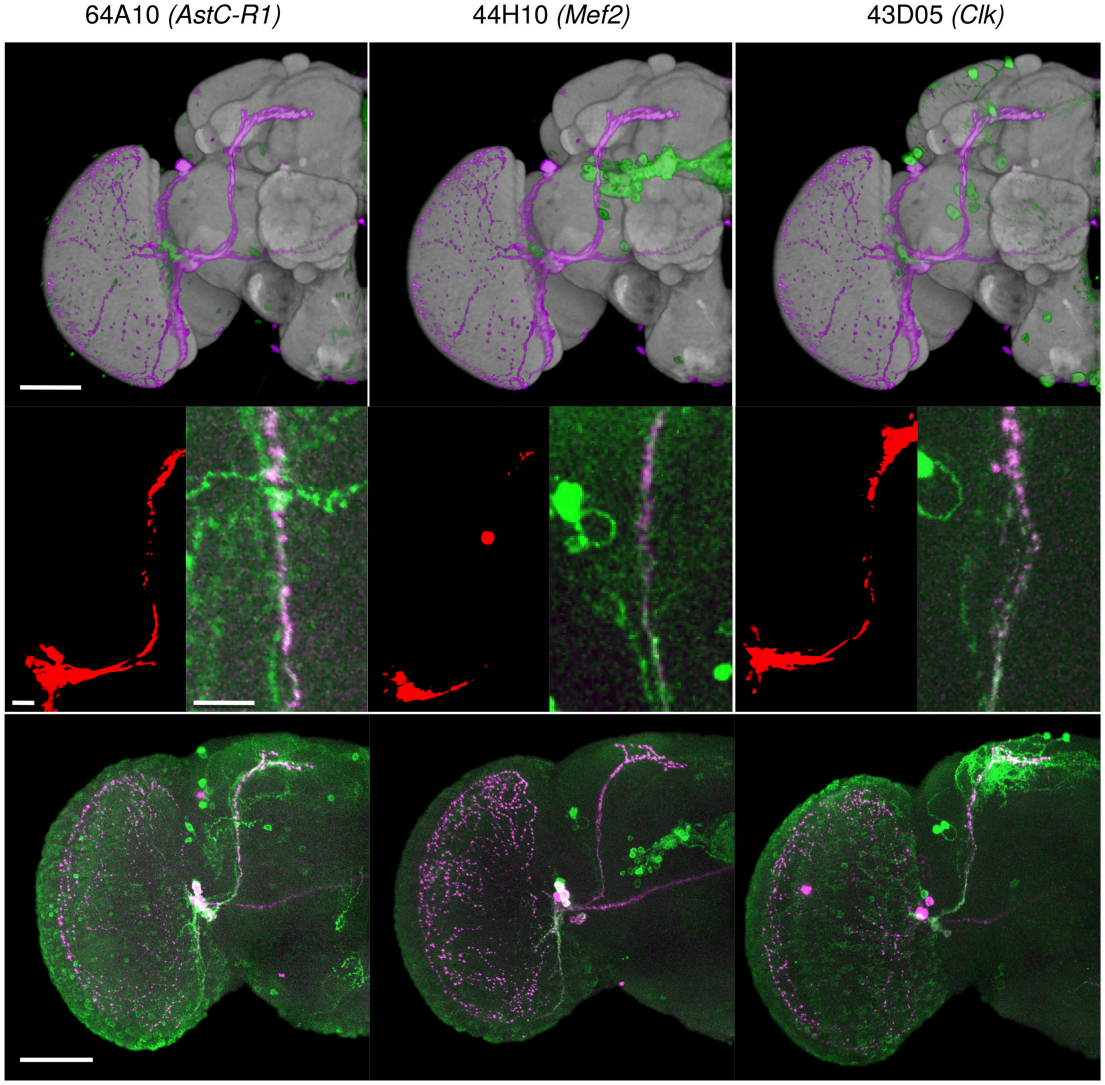
Three Janelia Farm lines identified with the BrainGazer 3D space query using PDF-expressing sLNv axonal tracts as a query template. **(Top)** Overlay of the PDF (*pink*) and GAL4-driven GFP (*green*) profiles registered on the standard brain. **(Middle)** Overlap (*red*) between the PDF profile 1 and the GAL4-driven GFP profile (left), and co-labeling of anti-PDF (*pink*) and anti-GFP (*green*) shown in the overlap region (right). *Bottom*: co-labeling of anti-PDF (*pink*) and anti-GFP (*green*) shown for one hemisphere. Numbers refer to Janelia Farm lines with associated gene names. Scale bars: 50 μm **(Top, Bottom)** and 10 μm **(Middle)**.

We tested the efficiency of our inter-sex template and registration procedure to retrieve gene expression profiles that intersect with each other. We used the anatomical 3D space query tool in BrainGazer software (Bruckner et al., 2009) to search the ~3,000 images of the Janelia Farm lines in the Tefor database with the typical axonal tracts of the PDF-expressing small ventral Lateral Neurons (sLNvs) (Beckwith and Ceriani, 2015). The search pattern was restricted to the most central part of the axonal tract in both hemispheres. Five individual *pdf-gal4 UAS-gfp* profiles were used independently to take into account the inter-individual variability of the axonal tracts (Figure 9). For each of the five sLNv profiles, 57–90 lines were recognized with more than 20% of overlap (arbitrary threshold) between the probe and the target profiles (Supplementary Table 1). The overlap was computed as the Dice coefficient between the binarized PDF and GAL4-driven GFP profiles inside the brush pattern. We restricted further analysis to the best fitting profiles by selecting lines whose overlap value was in the top 50 for at least three of the five sLNv profiles (Supplementary Figure 2; Supplementary Table 1). The obtained 42 profiles represented 36 individual genes, among which five clock genes (*Clk, cry, cwo, Mef2, Pdfr, per*) that are known to be expressed in the PDF neurons (Blanchard et al., 2010; Dubowy and Sehgal, 2017), thus validating the method. Visual inspection of the 42 profiles led us to select 16 lines for crosses with *UAS-gfp* line and immunolabeling of the progeny with anti-PDF and anti-GFP antibodies. Figure 10 shows the result for three of these lines that presented a clear co-expression of the GAL4-driven GFP and anti-PDF immunoreactivity. *Clk* and *Mef2* are two known clock genes, whereas the *AstC-R1* gene encodes an Allatostatin neuropeptide receptor whose expression profile is not characterized. Our search thus identified a new transcriptional enhancer that drives expression in the PDF cells and suggests that an Allatostatin signaling pathway plays a role in these clock neurons.

## 4. Discussion

Our study provides several new insights on the building of atlases of *Drosophila* adult brain. Based on an objective evaluation methodology, we first quantitatively established the importance of relying on group-wise atlases and provided guidelines for generating them. We then showed that state-of-the-art atlases, many of which have been built using the same computational procedure based on an affine transform followed by B-spline deformations, can be out-performed using alternative image registration algorithms that provide increased spatial accuracy. Lastly, we provided a web-based resource to access and query more than 3,000 GAL4 lines of the Janelia Farm FlyLight collection that we have registered onto a new average inter-sex atlas. The possibility of searching this database with the BrainGazer software allows to find axonal projections that are similar to any registered brain expression pattern and thus represents a unique tool to analyze neuronal circuits in the *Drosophila* brain.

Brain atlases can serve two purposes, automatic anatomical annotation and inter-individual comparison of image data. Using a single template for both tasks is advantageous, because it factorizes atlas building efforts and enforces the possibility of comparing results between different studies. Hence, atlases should be evaluated with regards to both objectives. Rohlfing et al. (2004) performed a detailed investigation of the performance of templates for the automated segmentation of bee brain images. To the best of our knowledge, the present study is the first to systematically evaluate insect brain templates for both tasks. Our results highlight that different individual templates may rank differently depending on the task and on the evaluation criterion. On the opposite, group-wise atlases systematically ranked best for both tasks. In addition, the median intensity template almost systematically ranked first compared to the average intensity template. Hence, using group-wise strategies not only brings robustness with increased accuracy and precision for each task, but it also brings more consistent performance across different tasks. The availability of robust and consistent atlases is relevant not only for the analysis of image collections, but also for the bridging between different atlases, which is required for comparing and integrating complementary data across different databases (Manton et al., 2014).

Several studies have evidenced sexual dimorphism in the organization of the adult *Drosophila* brain. On average, neuropil structures are larger in females than in males (Rein et al., 2002), which is assumed to be related to the overall larger body size of female flies, though localized regions involved in courtship behavior have been shown to be specifically enlarged in males (Cachero et al., 2010). At a smaller scale, sex-specific organization has also been shown in neuronal circuitry (Cachero et al., 2010). Several sex-specific templates have been built (Jenett et al., 2006, 2012; Chiang et al., 2011; Peng et al., 2011), resulting in the need for bridging transformations for inter-sex comparisons (Manton et al., 2014). Here, we built an inter-sex atlas that performed as well as sex-specific ones for most considered neuropil regions. Surprisingly, this template performed better for the automated segmentation of the protocerebral bridge in both sexes and, to a lesser extent, for noduli in males. These two structures were the two smallest regions considered in the present analysis (Rein et al., 2002) as well as the two regions with the smallest Dice segmentation scores with the sex-specific templates. In addition, the volumes of protocerebral bridge and of the noduli were respectively about 18 and 11% larger in the inter-sex atlas compared with the male-specific atlas. This was much above the increase observed for the other structures (average = +5%). For females, all structures but the protocerebral bridge (+2%) were smaller in the inter-sex atlas. The decreased sensitivity of the Dice coefficient to registration error when structure size increases could thus explain the structure- and sex-specific improved performance of the inter-sex template. We conclude that at least comparable performance is achieved by using an inter-sex rather than a sex-specific template. Our study thus objectively establishes the efficiency of using inter-sex templates for spatial normalization of *Drosophila* brain expression patterns and comparison between sexes. This result opens the perspective of simplifying the current landscape of *Drosophila* brain databases by removing the need of bridging sex-specific templates (Manton et al., 2014).

The need for average brain templates and atlases is increasingly recognized in the *Drosophila* community. In the absence of objective criteria, existing average brain atlases have been built using varying and arbitrary numbers of individual brains. For example, the *FCWB* template was built using 17 females and 9 males (Ostrovsky and Jefferis, 2014), the *Dmel* atlases were built using 14 females and 18 males (Ostrovsky et al., 2014), and 45 individuals were averaged in Yu et al. (2010). The selection of the number of brains in a statistical atlas should satisfy a compromise between statistical value and the human cost for manually annotating 3D images of reference brains. We provided here for the first time an objective study on the optimal number of individuals for a *Drosophila* adult brain atlas and showed that this optimum is task dependent. With about seven individuals, the average registration performance had already converged. Conversely, we observed a slower convergence for the automated segmentation task. This probably corresponded to the need for more individuals to compensate variability in the manual segmentation of neuropil regions. The difference between the convergence for segmentation and for registration suggests that a smaller number of individuals may be required when building an atlas for the purpose of comparing individual patterns rather than for the purpose of automatically annotating them. In any case, ten individuals were sufficient to reach convergence. This figure is at least twice below the number of individuals that have been used in several *Drosophila* average atlases until now. Our study thus suggests that these atlases may integrate more individuals than actually needed for optimal performance.

The comparison we performed with existing *Drosophila* brain templates showed that our group-wise atlas performed best for all neuropil structures and for both tasks. Different reasons may be invoked to interpret these observations depending on the considered alternative template. The *JFRC2010* template was built from a single individual. In light of the results of our comparisons between individual and group-wise atlases, the better performances of our atlas can be attributed to the higher accuracy and precision that are gained from the averaging approach. For the *Dmel* templates, the difference could not be fully explained by different acquisition conditions and the use of different neuropil staining antibodies. Indeed, a template generated in different and independent conditions to the ones used for the test brains still performed better than the three *Dmel* templates. Hence, the observed higher accuracy and precision of our template over the *Dmel* ones are probably due to the registration algorithms used to generate the respective templates (B-splines with smoothing regularization constraint vs. symmetric diffeomorphisms, for the deformation model; normalized mutual information vs. cross-correlation, for the registration metrics), in line with previous comparison on MRI human data (Klein et al., 2009). Investigating this hypothesis would require a detailed comparative analysis of algorithmic strategies, which was beyond the scope of the present study.

Three-dimensional brain atlases have been generated for several insect species other than *Drosophila*, including honey bee (Brandt et al., 2005), ant (Bressan et al., 2015), moth (Kvello et al., 2009), and desert locus (Kurylas et al., 2008). In most cases, these atlases were also developed for integrating image data across different individuals, for example for positioning individually labeled neurons into 3D maps of neuropil regions. However, as in *Drosophila*, these atlases have rarely been objectively evaluated for their use in automated registration or anatomical annotation of sample brain images (Rohlfing et al., 2004). Average brain atlases have been generated for several insect species (Brandt et al., 2005; Kurylas et al., 2008; Kvello et al., 2009; Heinze et al., 2013), using the Iterative Shape Averaging algorithm. The atlases that were out-performed in the present study by our group-wise atlas have also been generated using this algorithm. Because of the shared evolutionary history between insect classes, brain anatomical organizations exhibit common patterns and 3D images of neuropil-stained brains show similar contrasts. For all these reasons, we expect the group-wise registration algorithm introduced here for atlas building and the evaluation results we reported in *Drosophila* should also be relevant and beneficial to brain atlasing projects in many insect species.

Although several online databases are available to look for specific expression patterns in the brain (Bruckner et al., 2009; Chiang et al., 2011; Jenett et al., 2012; Milyaev et al., 2012), only the Virtual Fly Brain (VFB) website (http://www.virtualflybrain.org/site/vfb_site/home.htm; Milyaev et al. 2012) allows to analyze and compare GAL4 expression patterns through a brain atlas. A nice feature of VFB is the possibility of searching patterns that are similar to those of the FlyCircuit collection of single neuron labelings (Chiang et al., 2011; Costa et al., 2016). Our BrainBaseWeb 2.0 interface allows to analyze and compare more than 3000 GAL4 lines of the Janelia Farm FlyLight project that have been registered in our brain template. Importantly, the desktop version of BrainGazer allows to select in 3D any segment of an axonal tract or arborization that is displayed by a GAL4 line of our database and search the FlyLight collection for other lines sharing these particular axons or arborizations, as we describe here for the PDF-expressing small lateral neurons. Users can also perform similar searches through the BrainBaseWeb 2.0 interface, thanks to the newly added free form spatial query tool, which allows direct requests on image data without requiring prior segmentation into predefined anatomical regions. Providing superior flexibility in the definition of a query, this tool promises to become a very powerful feature in atlas-based research. In the near future, new collections of GAL4 (or other) lines will be added to our database. Future developments will allow users to switch expression patterns between brain templates (see Manton et al., 2014) and benefit from the tools developed by different projects to analyze specific neuronal populations and circuits.

## Author contributions

IA-C, JI, AJ, FR, and PA contributed to the design of the study and of experiments. TM and NM performed image acquisitions. IA-C performed numerical experiments. IA-C, TM, NM, AJ, FR, and PA analyzed the results. FS and KB implemented the database and its web interface. IA-C, TM, KB, AJ, FR, and PA wrote the paper. All authors approved the manuscript.

### Conflict of interest statement

The authors declare that the research was conducted in the absence of any commercial or financial relationships that could be construed as a potential conflict of interest.

## Supplementary material

Supplementary Figure 1 3D renderings of the 14 regions used for quantitative evaluation of atlas performances in segmentation and registration tasks. The 14 regions shown here were extracted from the atlas of Ito et al. (2014) that has been registered onto the group-wise inter-sex atlas (available from http://fruitfly.tefor.net).

Supplementary Figure 2 Selected lines from the Janelia Farm collection showing an overlap value with the search pattern ranking among the first 50 for at least three of the five PDF profiles. **(Left)** GAL4-driven GFP profile registered on the standard brain. **(Right)** overlap between the first PDF profile and the GAL4-driven GFP profile. Numbers refer to Janelia Farm lines with associated gene names. Scale bar: 20 μm.

Supplementary Table 1 Results of the 3D space query for each of the five PDF profiles. Overlap values are indicated for each Janelia Farm line and the corresponding gene name (FlyBase nomenclature) is indicated for the overlap values ranking among the first 50 for at least three of the five PDF profiles (*blue*). Bold names correspond to the three lines shown in Figure 10.

Supplementary Movie 1 Animated rendering of the group-wise inter-sex atlas. Successively: nc82 template image (2D sections then 3D volume rendering, opaque then transparent); label image (3D surface rendering of anatomical regions, defined following Ito et al. 2014); six registered patterns of GAL4-GFP expression (3D surface rendering of intensity-thresholded pattern images); same patterns (left half of the brain) with the anatomical regions (right half of the brain).
