# Supplementary material for: A Statistically Representative Atlas for Mapping Neuronal Circuits in the Drosophila Adult Brain
Source: Front Neuroinform. 2018 Mar 23;12:13. doi: 10.3389/fninf.2018.00013 (PMC5876320; doi:10.3389/fninf.2018.00013)
Supplement: Supplementary Figure 1 — 3D renderings of the 14 regions used for quantitative evaluation of atlas performances in segmentation and registration tasks. The 14 regions shown here were extracted from the atlas of Ito et al. (2014) that has been registered onto the group-wise inter-sex atlas (available from http://fruitfly.tefor.net). [file Image1.PDF]

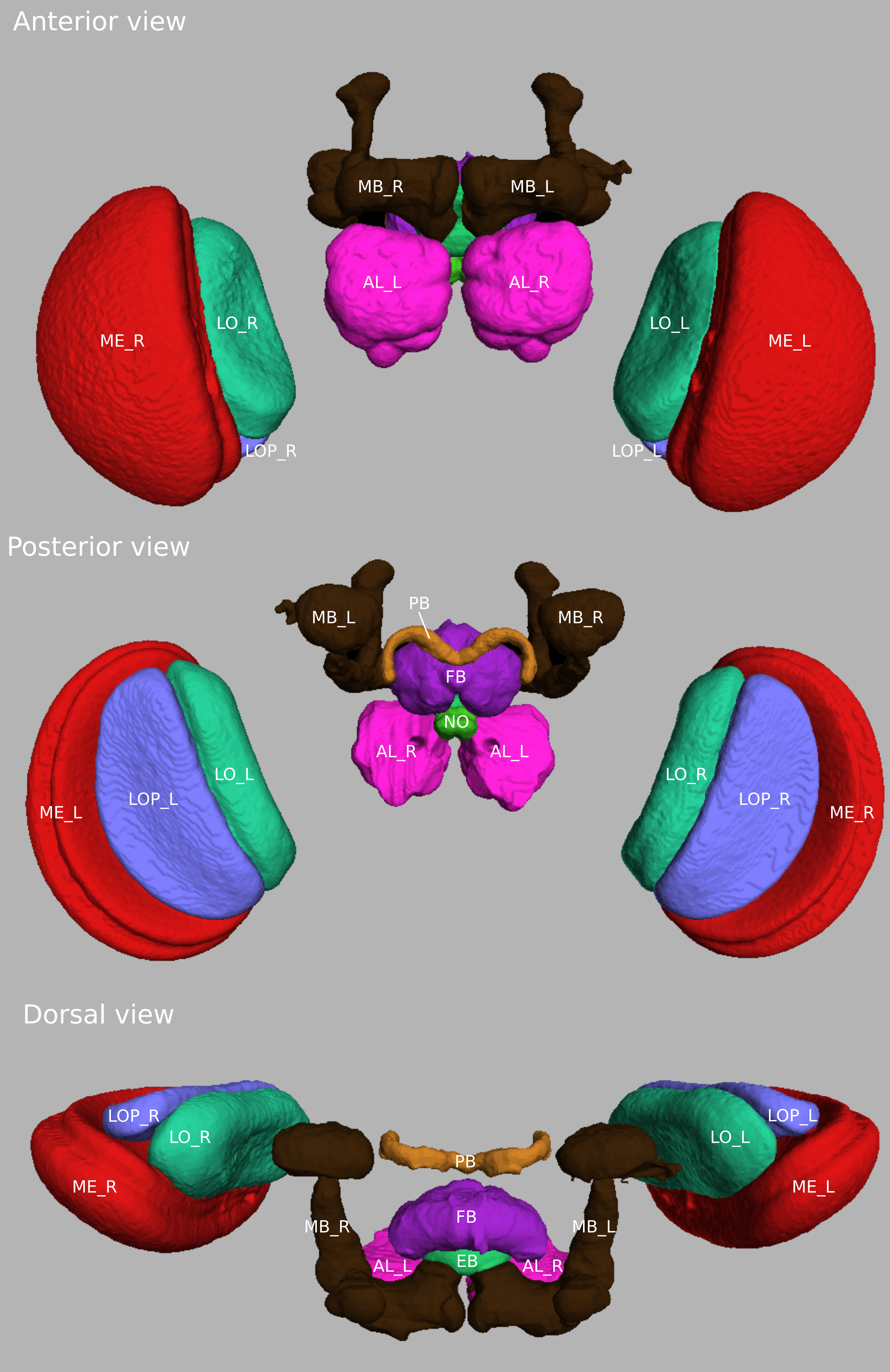

AL_L: Antennal lobe (left), AL_R: Antennal lobe (right), EB: Ellipsoid body, FB: Fan-shaped body, NO: Noduli, PB: Protocerebral bridge, MB_L: Mushroom body (left), MB_R: Mushroom body (right), LO_L: Lobula (left), LO_R: Lobula (right), LOP_L: Lobula plate (left), LOP_R: Lobula plate (right), ME_L: Medulla (left), ME_R: Medulla (right)
