# Supplementary material for: A Statistically Representative Atlas for Mapping Neuronal Circuits in the Drosophila Adult Brain
Source: Front Neuroinform. 2018 Mar 23;12:13. doi: 10.3389/fninf.2018.00013 (PMC5876320; doi:10.3389/fninf.2018.00013)
Supplement: Supplementary Figure 2 — Selected lines from the Janelia Farm collection showing an overlap value with the search pattern ranking among the first 50 for at least three of the five PDF profiles. (Left) GAL4-driven GFP profile registered on the standard brain. (Right) overlap between the first PDF profile and the GAL4-driven GFP profile. Numbers refer to Janelia Farm lines with associated gene names. Scale bar: 20 μm. [file Image2.PDF]

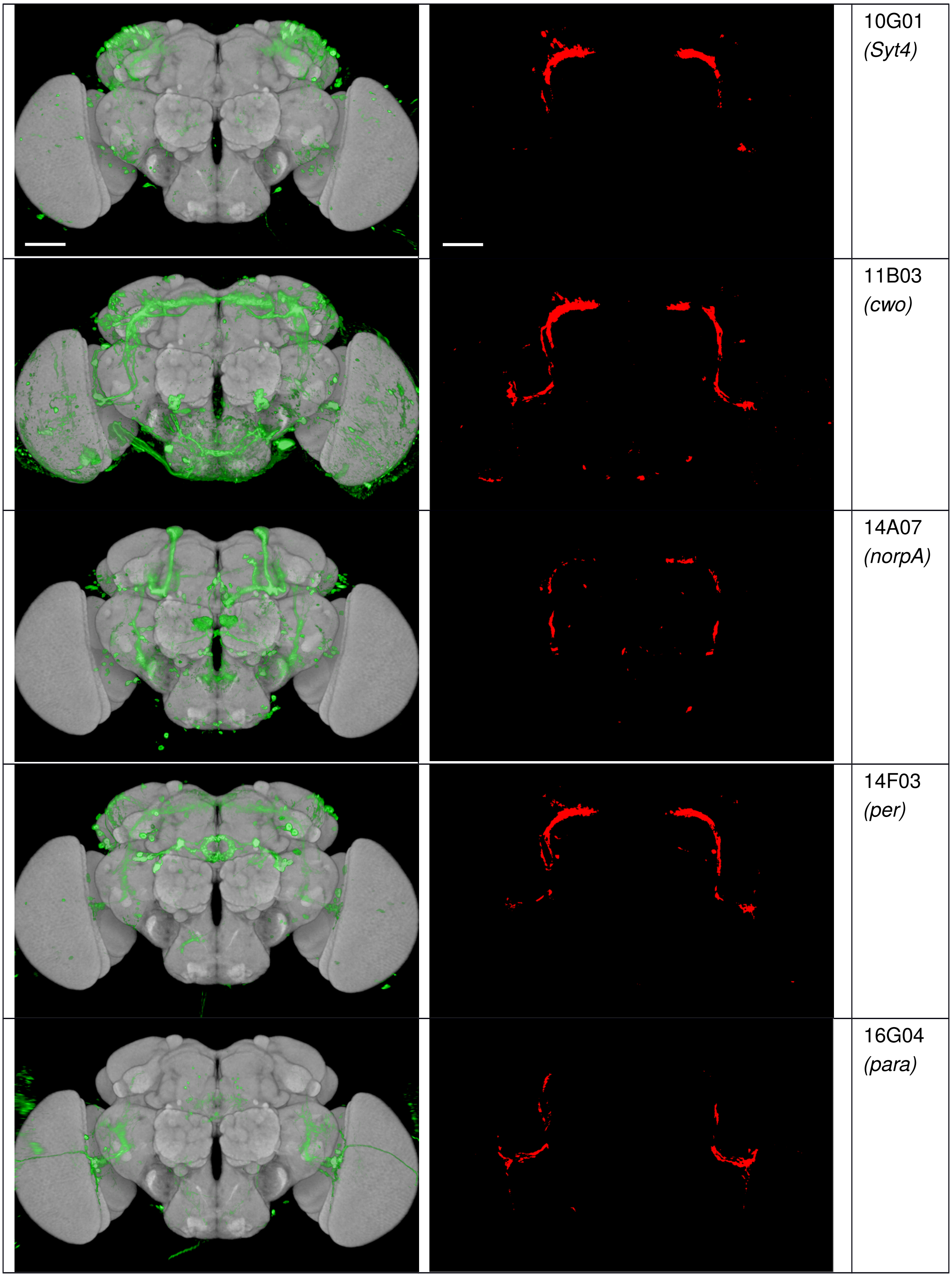

10G01
(Syt4)
11B03
(cwo)
14A07
(norpA)
14F03
(per)
16G04
(para)

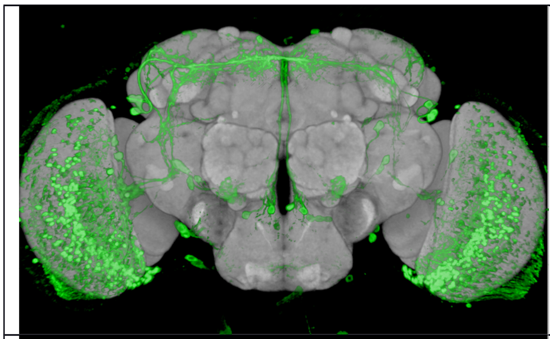	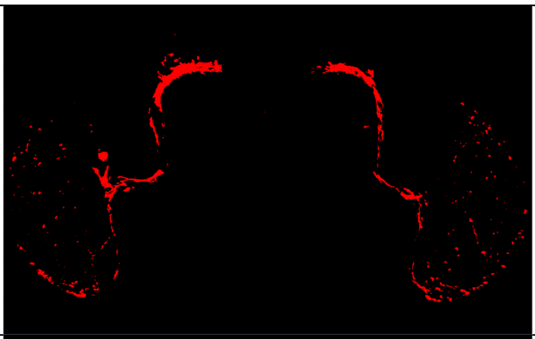	18F07 *(Pdfr)*
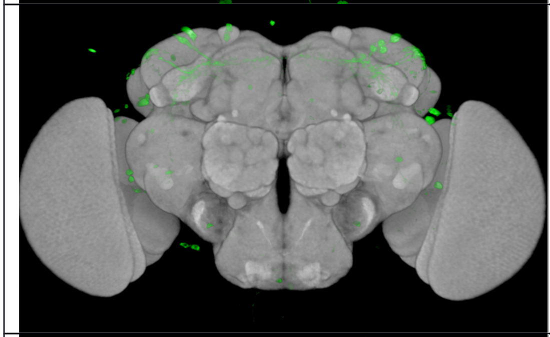	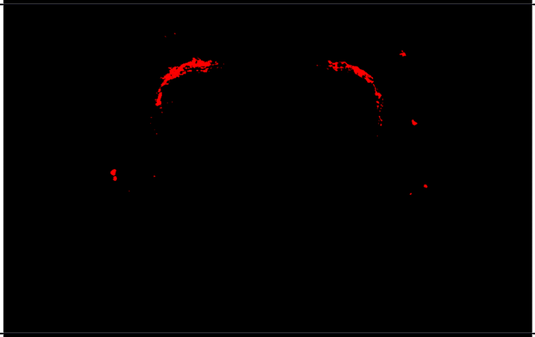	18H11 *(Pdfr)*
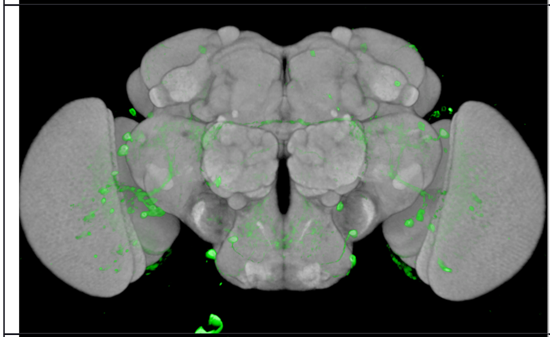	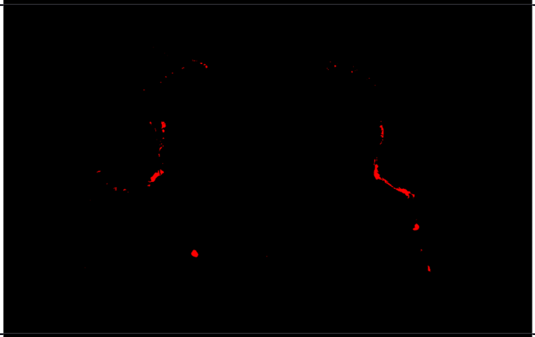	20B10 *(Trpm)*
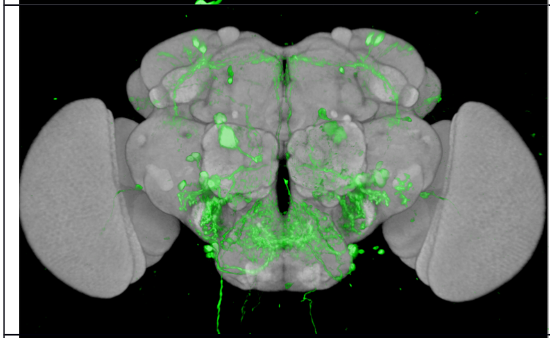	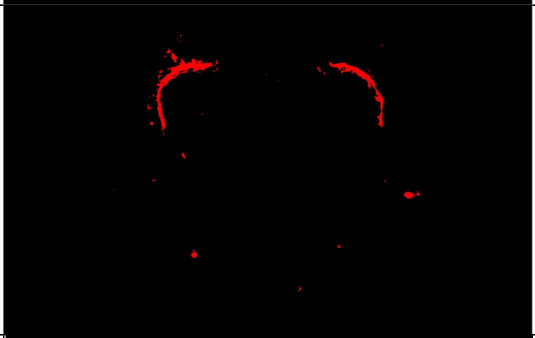	20G07 *(Trpm)*
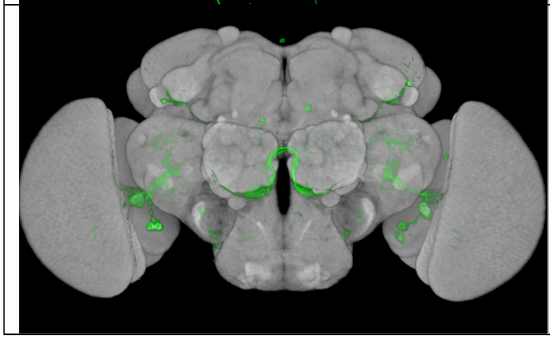	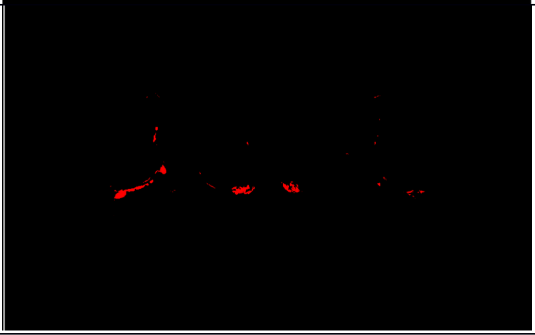	$22^{E}12$ *(Mip)*

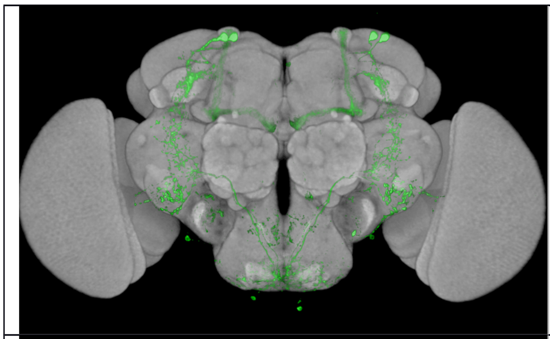	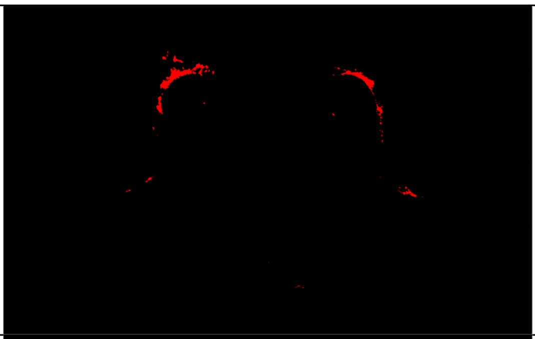	23^{E}05 *(hec)*
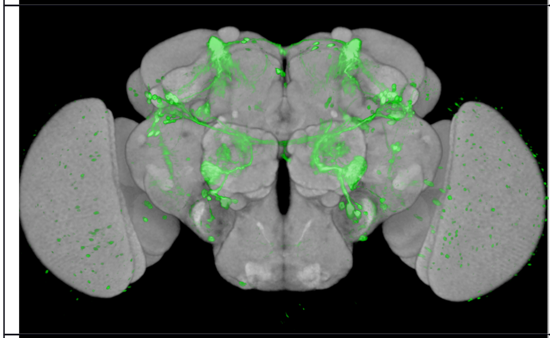	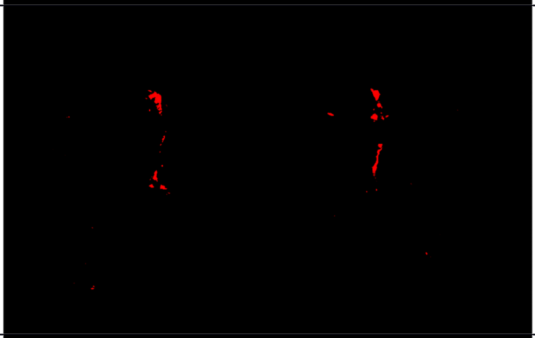	24H08 *(Dh31-R1)*
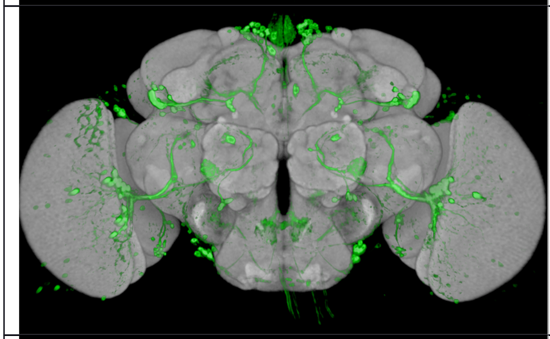	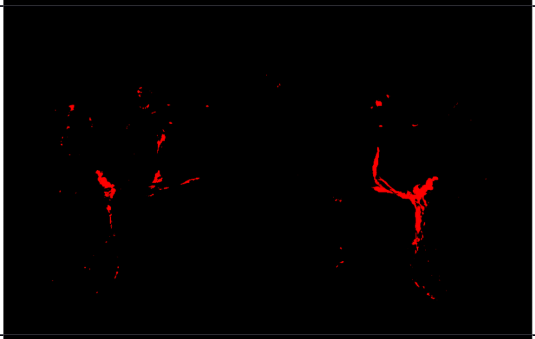	25F06 *(tn)*
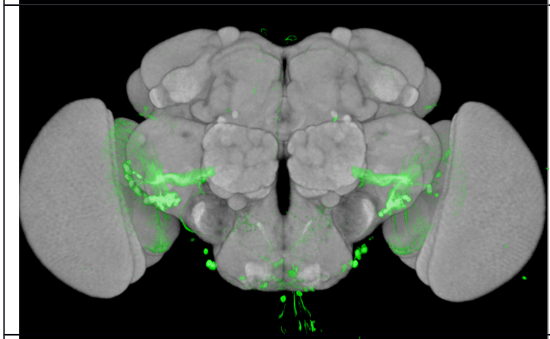	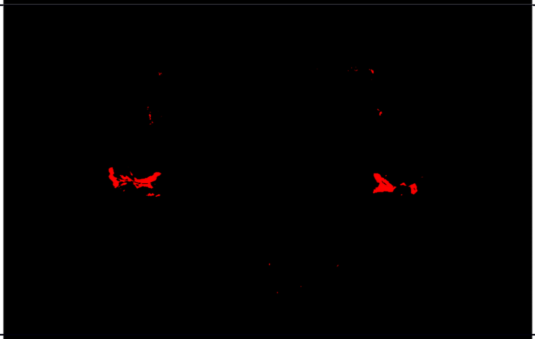	26G09 *(grn)*
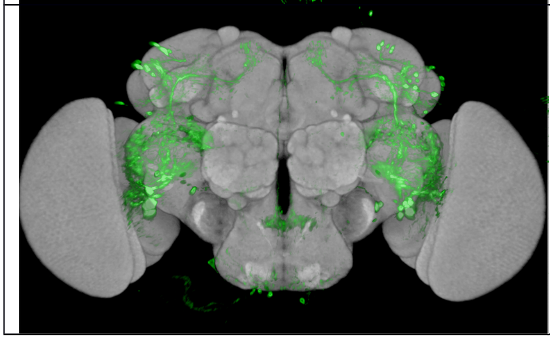	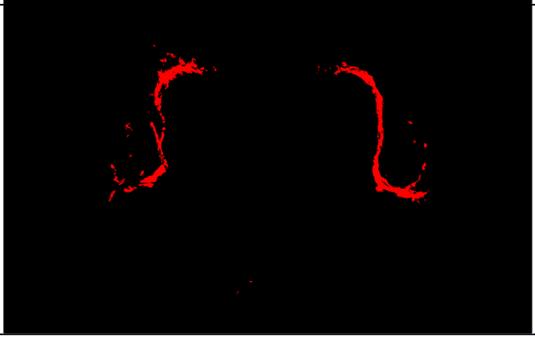	31B01 *(CadN)*

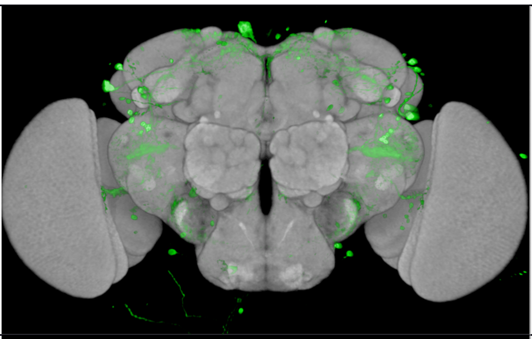	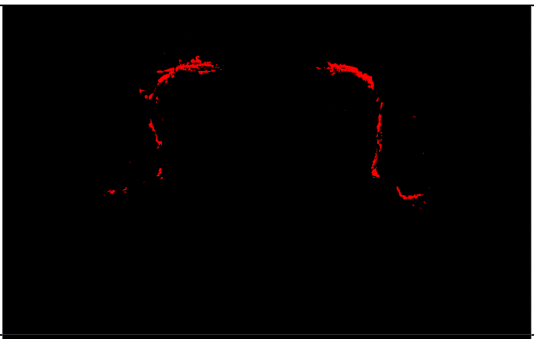	31C03 *(CadN)*
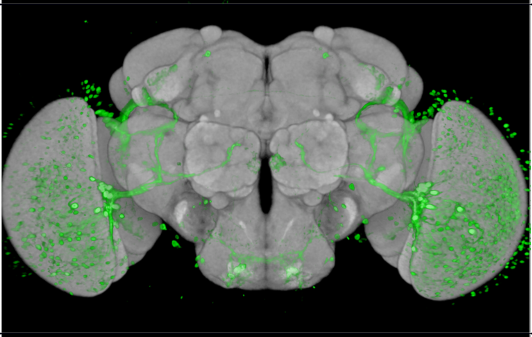	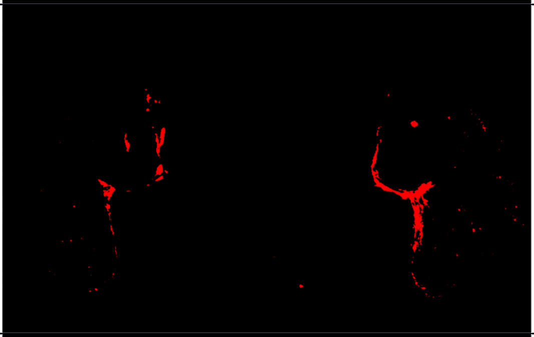	32D06 *(CadN)*
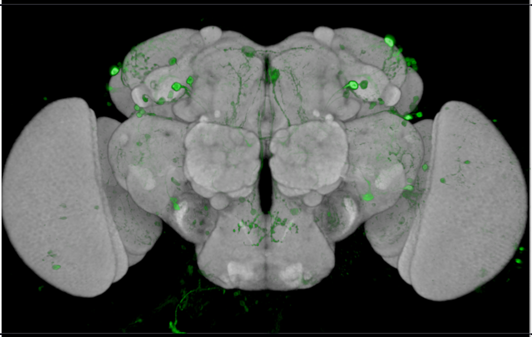	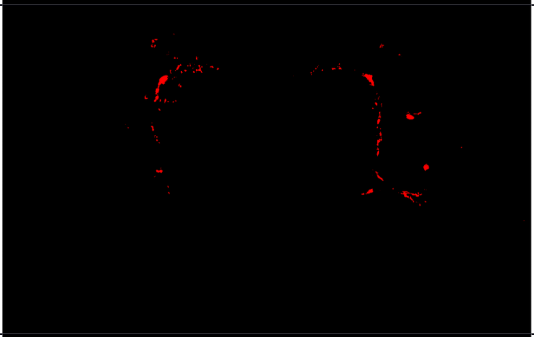	35F03 *(Hr38)*
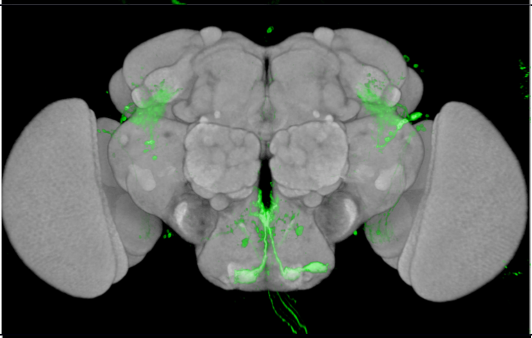	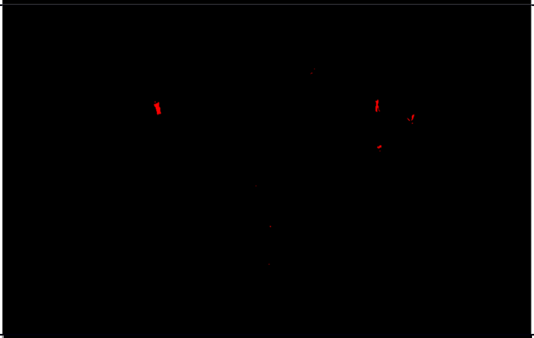	37F05 *(rdgA)*
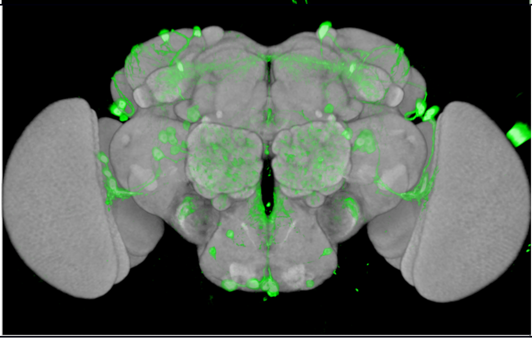	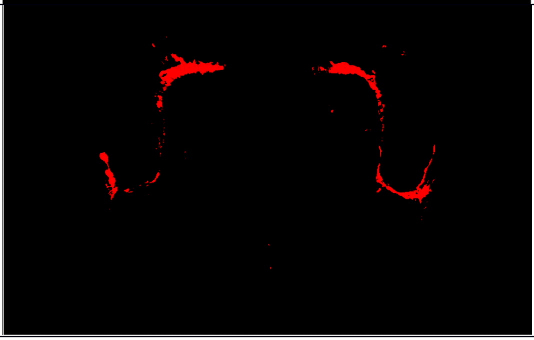	43D05 *(Clk)*

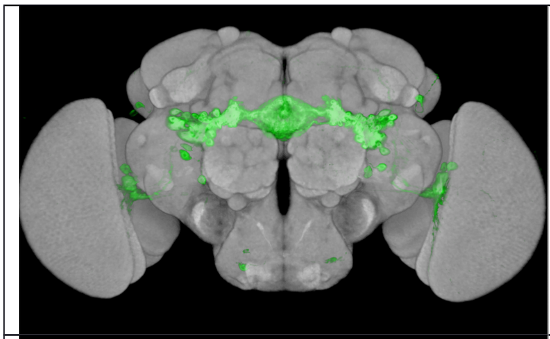	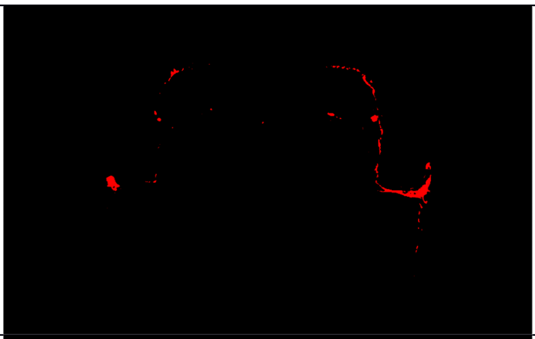	44H10 *(Mef2)*
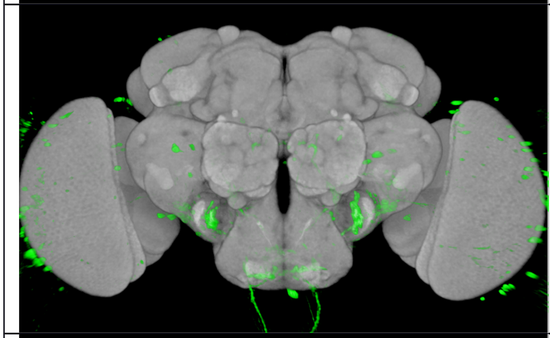	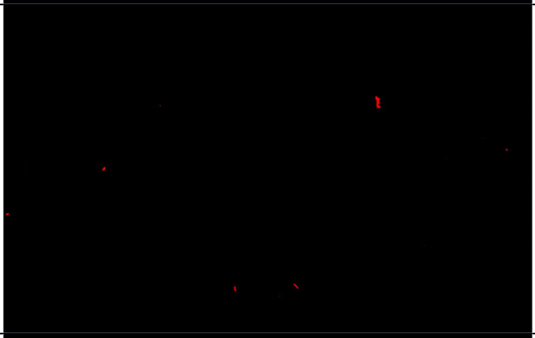	53A02 *(klg)*
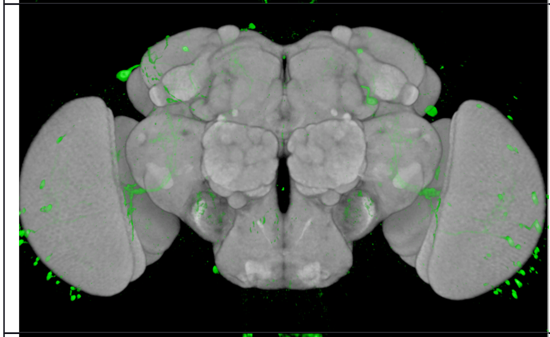	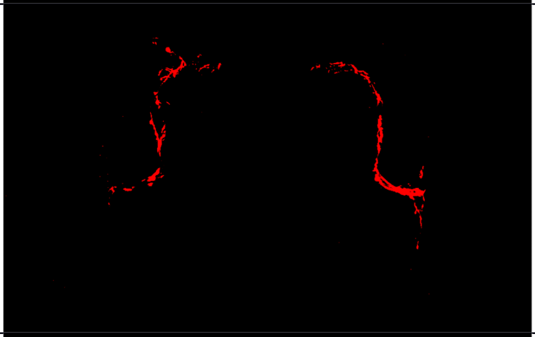	53B03 *(5-<br>HT1A)*
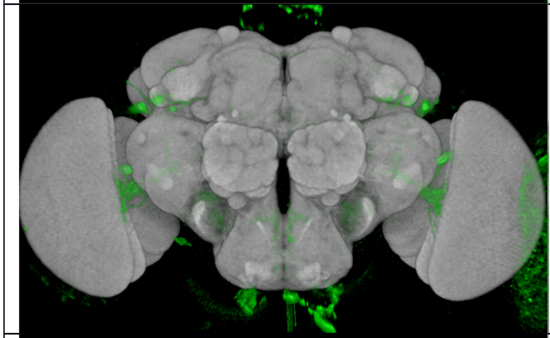	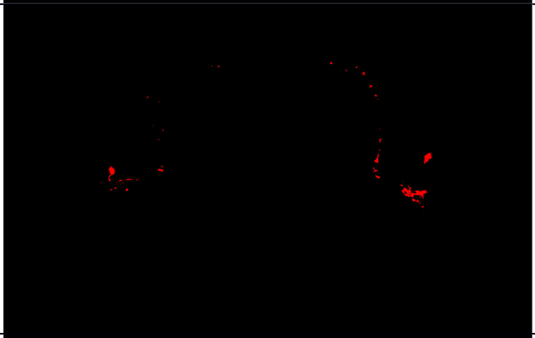	54D11 *(CG114<br>48)*
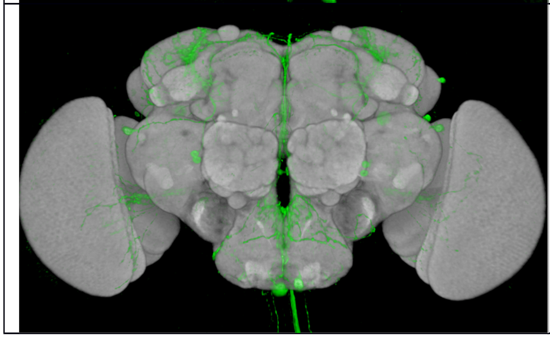	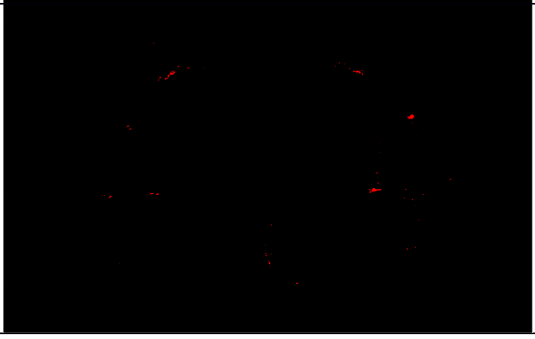	55A10 *(CG147<br>62)*

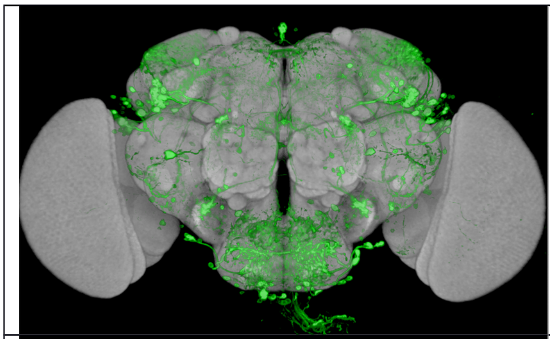	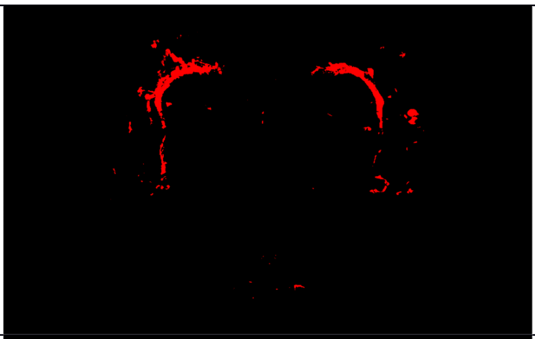	64A07 *(TkR86 C)*
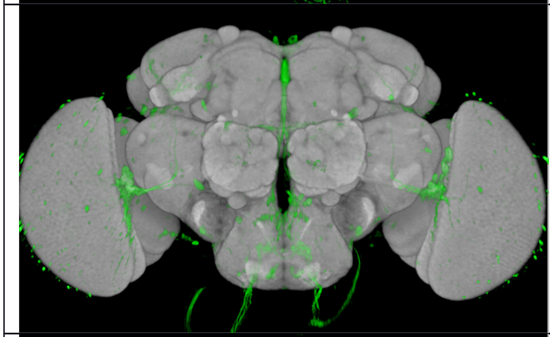	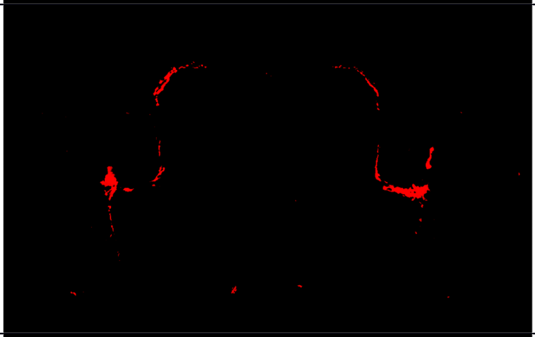	64A10 *(AstC-R1)*
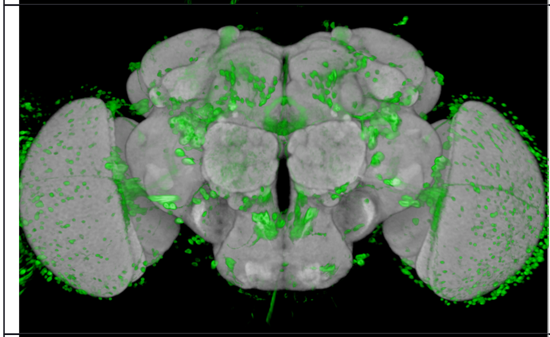	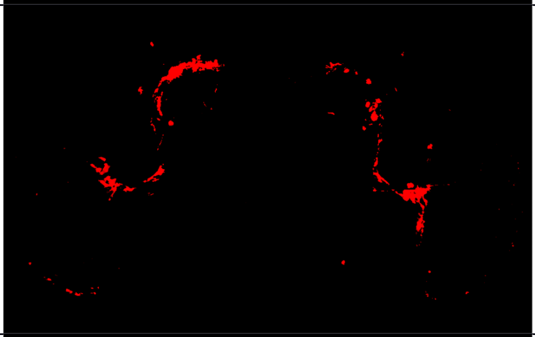	64C10 *(cry)*
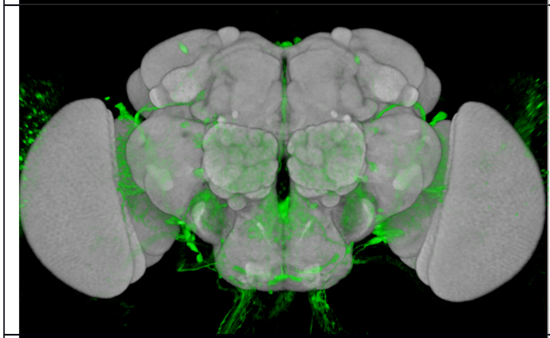	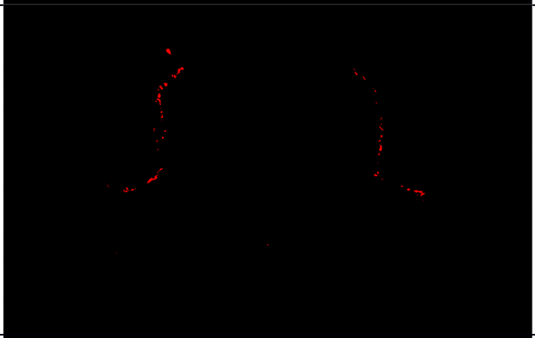	65B02 *(AstC-R1)*
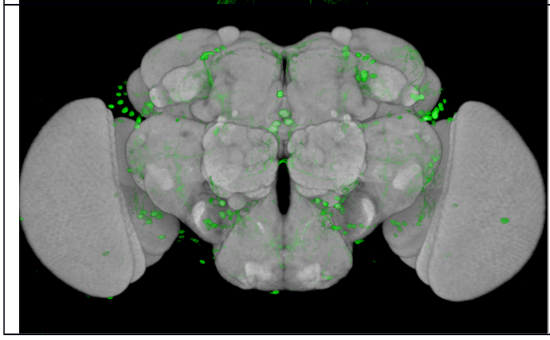	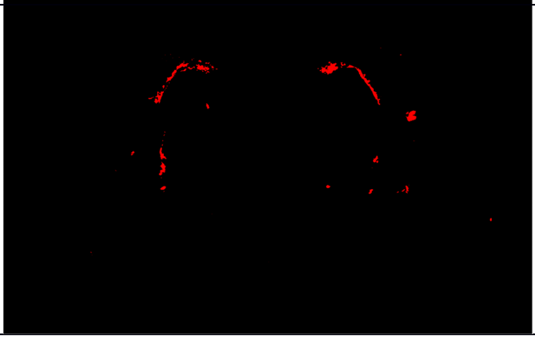	65D09 *(AstC-R2)*

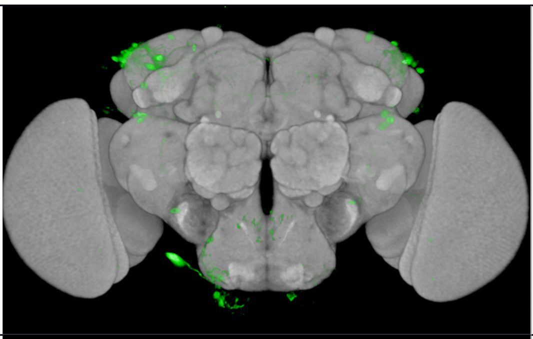	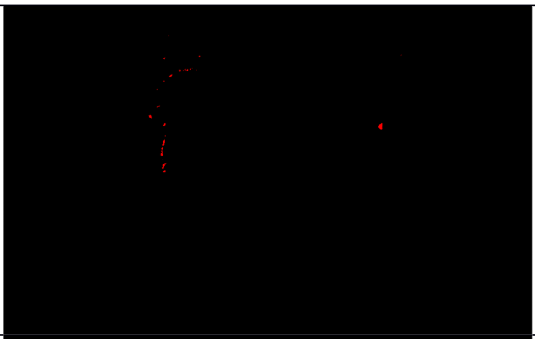	65G02 *(CASK)*
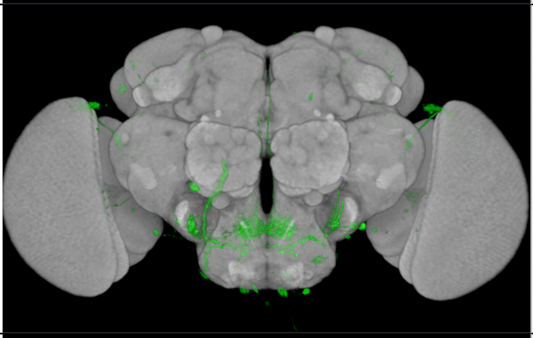	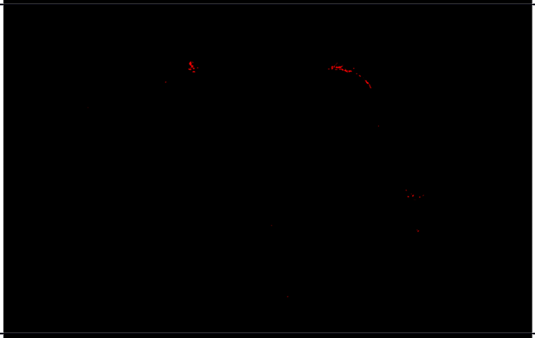	67F03 *(AstC)*
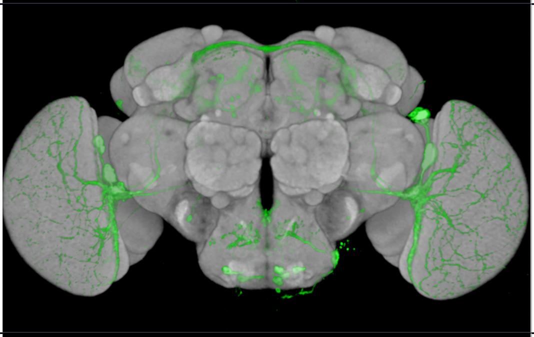	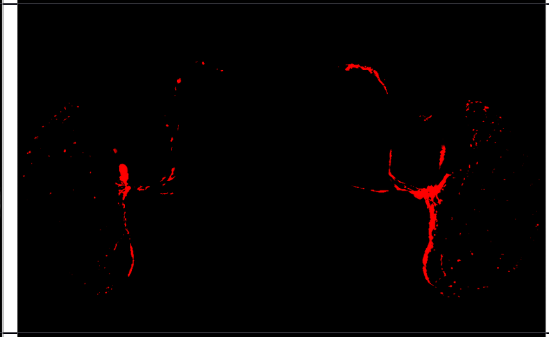	71G01 *(Vsx2)*
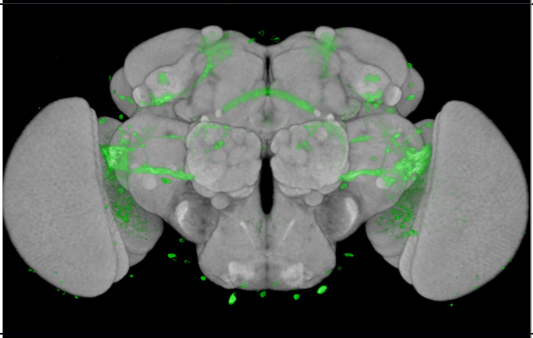	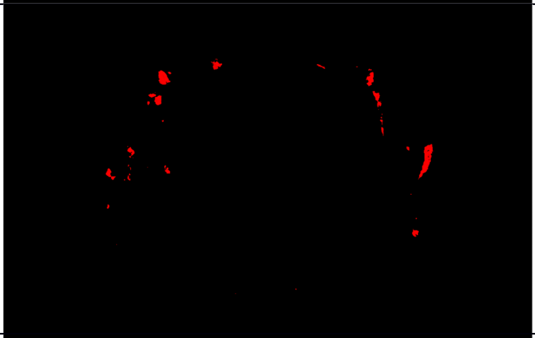	72G06 *(CG317 14)*
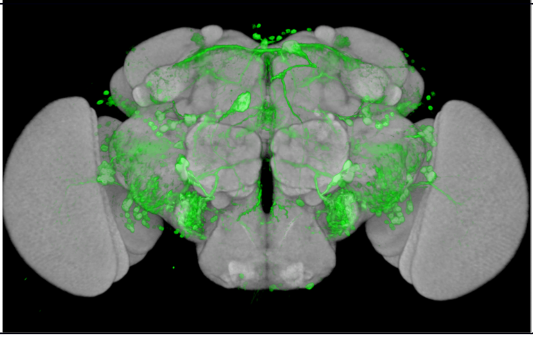	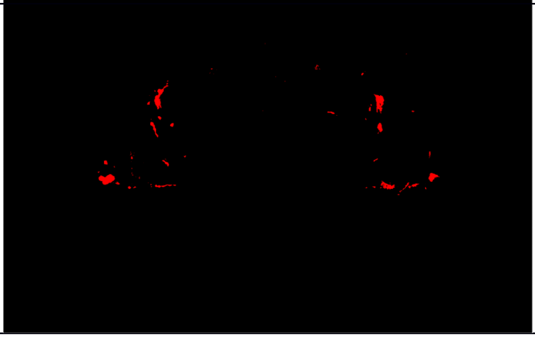	76F12 (amon)

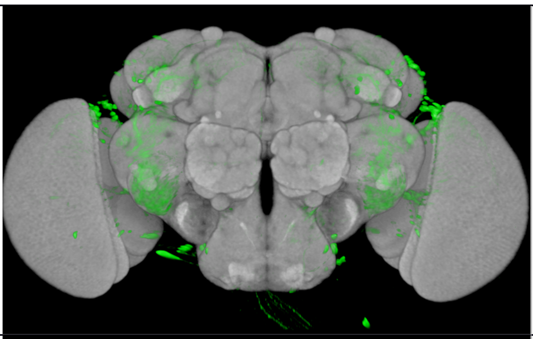	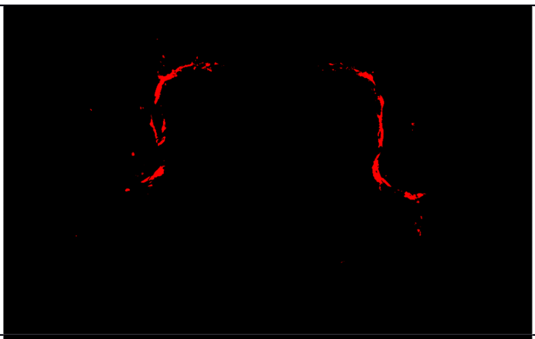	77H08<br>*(CG18599)*
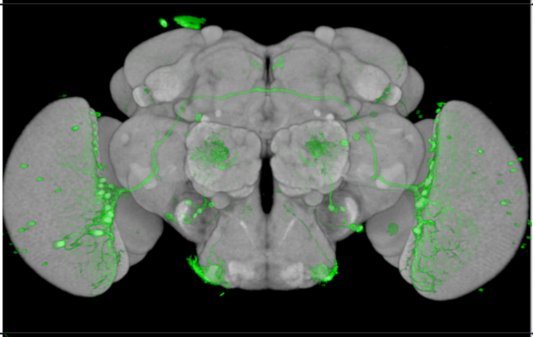	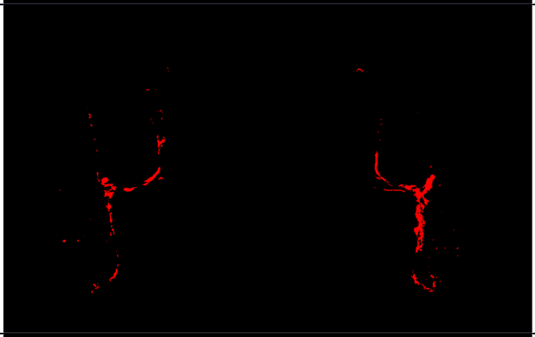	78H08<br>*(Vsx2)*
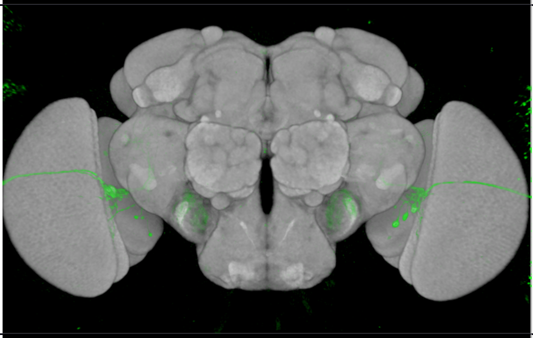	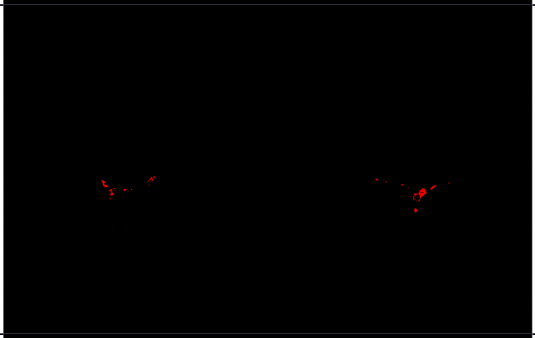	79A08<br>*(esn)*
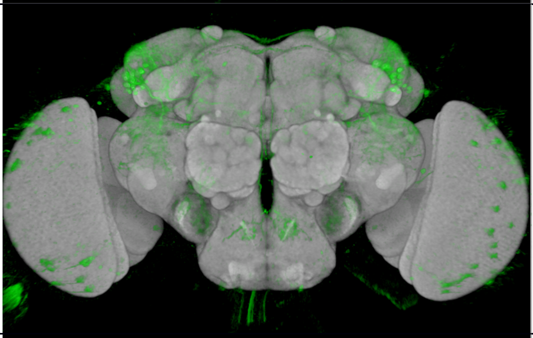	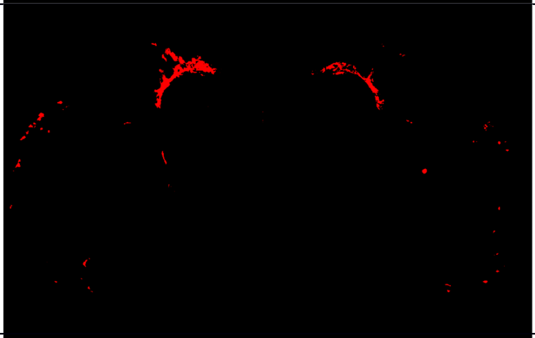	80C12<br>*(CG7587)*
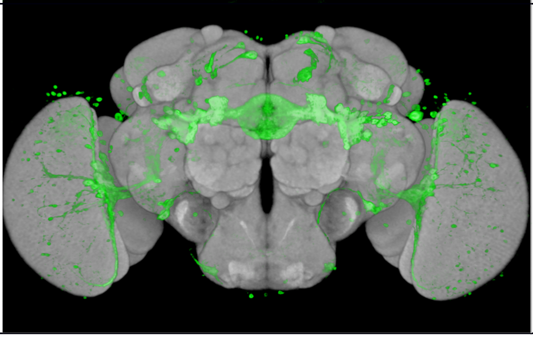	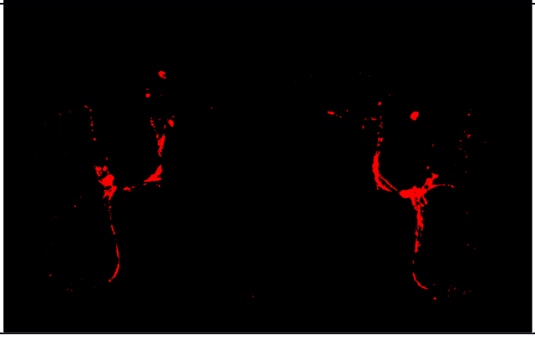	82H12<br>*(Octβ3R)*

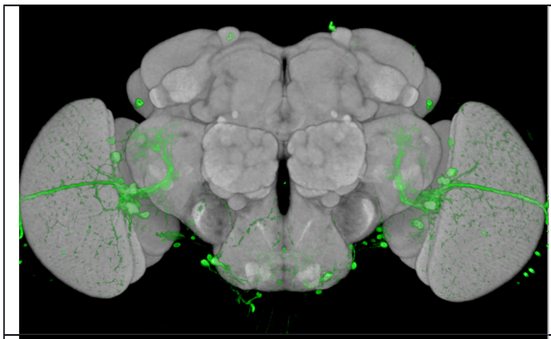	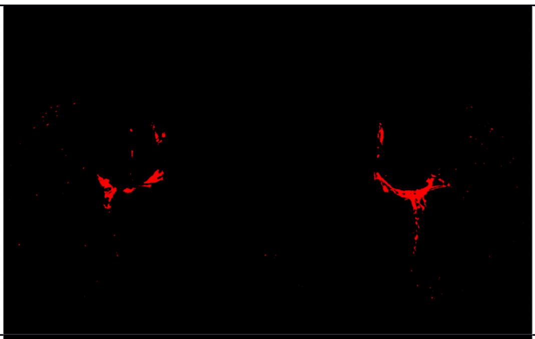	85G07<br>*(shn)*
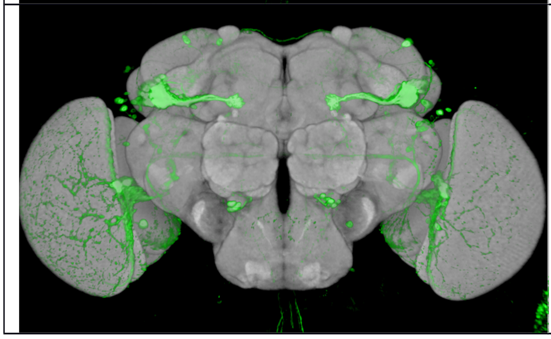	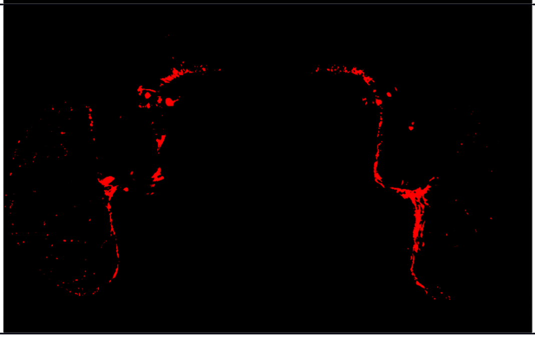	92H07<br>*(CG31345)*
